# 90‐Day Subchronic Toxicological Evaluation of a Zylaria in Sprague Dawley Rats

**DOI:** 10.1155/jt/6989296

**Published:** 2026-04-12

**Authors:** Devanand Shanmugasundaram, Richard Anthony Wang

**Affiliations:** ^1^ Department of Compliance and Operations (Toxicology), Vedic Lifesciences Pvt. Ltd., Mumbai, Maharashtra, India; ^2^ NuLiv Science, Brea, 92821, California, USA

**Keywords:** histopathology, Sprague Dawley rats, toxicity, weight, Zylaria

## Abstract

**Objective:**

Zylaria comprises a blend of botanical components, including *Xylaria Nigripes* (mycelium), *Cuscuta Chinensis* (seed) and *Panax Notoginseng* (root). This study aimed to evaluate the potential toxicity of Zylaria when administered orally (via gavage) to Sprague Dawley (SD) rats for 90 days continuously and examine any delayed toxicity after a minimum recovery period of 28 days post‐treatment cessation.

**Methods:**

One hundred SD rats of both sexes were divided into six study groups: four main groups with 10 rats of each sex receiving different doses of Zylaria (0, 1000, 2750 and 4500 mg/kg body weight per day) and two recovery groups with five rats of each sex receiving either the vehicle control (Milli‐Q water) or high‐dose Zylaria. Throughout the study, animals were monitored daily for general behaviour, body weight fluctuations and clinical signs. Upon completion of the treatment period, haematological, coagulation, clinical chemistry, thyroid hormone analyses and histopathological examination of organs were conducted.

**Results:**

Oral administration of Zylaria at tested concentrations did not induce any adverse events on general health, body weight, relative organ weights, or haematological, coagulation, clinical chemistry, and thyroid hormone parameters. Histopathological examination demonstrated no significant structural alterations in organs, even in animals treated with high doses of Zylaria. No test item–related effects were observed during the 28‐day recovery period after cessation of the treatment.

**Conclusion:**

The study concluded that Zylaria treatment for 90 days does not lead to toxicity, even at doses up to 4500 mg/kg bw/day, indicating its safety for use.

## 1. Introduction

Toxicity testing is crucial for assessing the safety of new drugs before human use. It involves evaluating potential hazards by administering the drug to various animal species over time and monitoring for clinical, haematological, biochemical and histopathological abnormalities. This process includes detailed statistical analysis and data interpretation to ensure the drug’s safety.

[1] Zylaria formulation includes *Xylaria Nigripes* (mycelium), *Cuscuta Chinensis* (seed) and *Panax Notoginseng* (root). Each component offers distinct medicinal properties that have been traditionally recognised and scientifically investigated. *Xylaria nigripes,* a medicinal mushroom, is used for treating insomnia, trauma, and as a diuretic and nerve tonic [2]. It has demonstrated antioxidant [3, 4], immunomodulatory [5] and hepatoprotective activities [6]. Lai et al. conducted a 90‐day toxicity study in rats with *X. nigripes* (XNE) aqueous extract, which showed no significant toxicity even at doses up to 100‐fold (2000 mg/kg body weight/day) higher than recommended daily intakes [7]. *Cuscuta chinensis*, a key herb in traditional Chinese medicine [8], exhibits anticancer [9], anti‐mutagenic [10], antidiabetic [11], antioxidant, hepatoprotective [12], antiaging [13], antiosteoporotic [14], genoprotective [15], neuroprotective [16], memory‐enhancing [17], antinociceptive and anti‐inflammatory properties [18]. Maimaiti et al. investigated the acute and sub‐acute toxicities of *C. chinensis* water extract (CLW) in ICR mice. The acute toxicity study revealed an LD50 of over 5000 mg/kg, and a sub‐acute study showed no mortality and severe toxicity at doses below 1250 mg/kg, indicating CLW is safe at these doses [19]. *Panax notoginseng*, another cornerstone of traditional Chinese medicine, offers various clinical benefits, including anti‐inflammatory, antioxidant, antiplatelet aggregation and neuroprotective effects [20]. Toxicity studies by Chan PC et al. confirm the safety profile in both short‐term (2‐week repeated dose toxicity studies, 3‐month subchronic studies) and long‐term administration (2‐year chronic toxicity and carcinogenicity studies) [21]. While individual toxicity studies on these plant components suggest safety, it is essential to evaluate the safety of the Zylaria formulation as a whole.

However, preliminary data from a 14‐day repeated dose oral toxicity study conducted on rats by Vedic Lifesciences indicates its tolerability at a dose of 5000 mg/kg bw; comprehensive long‐term toxicity studies specifically targeting the Zylaria formulation itself have not yet been conducted. Therefore, this study aimed to address this critical gap by systematically evaluating the potential toxicity of Zylaria when administered orally (via gavage) to Sprague Dawley (SD) rats for 90 days continuously and to examine any delayed toxicity after a minimum recovery period of 28 days post‐treatment cessation.

## 2. Materials and Methods

### 2.1. Reagents and Kits

Kits and reagents for clinical chemistry, coagulation and haematology parameters were obtained from Randox Laboratories (United Kingdom), Diagnostica Stago S.A.S (France) and Mindray Animal Care (China), respectively. ELISA kits for T3, T4 and TSH were procured from Wuhan Huamei Biotech Co., Ltd., P.R. China (Catalogue No. T3‐CSB‐E05115r; T4‐CSB‐E05082r; CSB‐E05115r).

### 2.2. Test Material Preparation

The mycelium powder of *Xylaria nigripes*, along with extracts of *Panax notoginseng* and *Cuscuta chinensis*, was blended, sieved through a 100‐mesh screen and filtered through a magnetic filter to obtain the final product (Zylaria powder). This Zylaria powder was sent for encapsulation at a cGMP‐compliant facility [Figure S1a–S1d].

*Cuscuta Chinensis* (seed), *Panax notoginseng* (root) and Zylaria powder have been screened for high‐performance liquid chromatography (HPLC) profiling at Alkemist Labs, USA [Figure S2a–S2c]. In addition, Zylaria has been subjected to analytical screening for total flavonoids (%), polysaccharides (%) at Eurofins Technology Service (Guangzhou) Co., Ltd, China, and γ aminobutyric acid (mg/kg) at SGS‐CSTC Standards Technical Services (Shanghai) Co., Ltd., China [Figure S3a‐S3c].

### 2.3. Animal Ethical Approval and Welfare

The research complied with all the relevant national regulations and institutional policies for the care and use of animals. The study was conducted in India following the Committee for the Purpose of Control and Supervision of Experiments on Animals (CPCSEA) guidelines, the Institutional Animal Ethics Committee (IAEC) and Vedic Lifesciences Standard Operating Procedures (Approval No. VIP‐IAEC‐356‐2023). These procedures align with the American Association for Accreditation of Laboratory Animal Care (AAALAC), USA‐accredited guidelines [22].

Adult (∼7–8 weeks old) male and female SD rats were procured from a CPCSEA‐approved vendor. The animals were housed under experimental conditions (temperature 19.5–23.8°C, relative humidity 45%–69% and 12 h alternate light/dark cycle) with ad libitum access to commercial pellet feed and reverse osmosis water [22].

### 2.4. Study Design

The study followed the OECD 408 guideline for the testing of chemicals [23]. One hundred rats (50 males and 50 female rats) were divided into six groups: four main groups (10 animals/sex) and two recovery groups (five animals/sex). At the start of acclimatization, the rats were 7–8 weeks old, with males weighing 301.71–371.97 g and females weighing 168.98–144.28 g. The weight variation at randomisation was within ± 20% of the mean body weight for each sex and group.

The animals were allocated to six groups as follows:

 Group 1 (G1): vehicle control (0 mg/kg bw/day);
 Group 2 (G2): low‐dose Zylaria (1000 mg/kg bw/day);
 Group 3 (G3): mid‐dose Zylaria (2750 mg/kg bw/day);
 Group 4 (G4): high‐dose Zylaria (4500 mg/kg bw/day);
 Group 1R (G1R): vehicle control recovery (0 mg/kg bw/day);
 Group 4R (G4R): high dose of Zylaria recovery (4500 mg/kg bw/day).

Oral administration is convenient, safe and allows for the voluntary cooperation of animals. For safety assessment, it mimics the intended route of administration in humans [24]. A dose volume of 10 mL/kg bw was administered to all groups. Zylaria in Milli‐Q water was administered orally to animals in both sexes at the doses of 1000 (G2), 2750 (G3) and 4500 (G4, G4R) mg/kg bw/day for 90 days. Similarly, the male and female vehicle control groups (G1 and G1R) received Milli‐Q water for 90 consecutive days. A 14‐day dose range–finding study was conducted to determine optimal doses for the 90‐day study trial (however, the data were unpublished).

### 2.5. Observations

All animals were administered with test item once daily (QD) by oral gavage throughout the 90‐day treatment period. Animals were observed once daily for clinical signs and twice daily for morbidity and mortality throughout the study period. Observations included changes in skin, fur, eyes and mucus membranes, as well as autonomic activity, gait, posture and behaviour. A functional observation battery (FOB) was conducted during the final week of treatment across all main and recovery groups. Assessments included observations of the home cage, handling, open field, sensory reactivity measurement, nervous and muscle function evaluation, foot splay, grip strength and motor activity. Ophthalmological examinations using a Welch–Allyn direct ophthalmoscope, with mydriasis induced by 1% tropicamide, were performed before and after treatment in the control (G1) and high‐dose (G4) groups and during the last week of the recovery period. Body weight and feed consumption were recorded weekly throughout the study period [22].

### 2.6. Clinical Pathology

At the end of the experiment, blood samples were collected from the retro‐orbital plexus under mild isoflurane anaesthesia. Samples were drawn into tubes containing 10% dipotassium ethylene diamine tetra acetic acid (K2‐EDTA) for haematology, sodium citrate for coagulation assessment and plain tubes for clinical chemistry.

Haematological parameters (e.g., white blood cell count (WBC), red blood cell count (RBC), haemoglobin (HGB), haematocrit (HCT), etc.) were analysed using a Mindray BC‐5000 VET haematology analyzer. Coagulation was assessed by a Stago Max coagulation analyzer [22]. Blood smear examination was conducted for differential leukocyte and reticulocyte counts. Clinical chemistry parameters were analysed using a Randox Daytona Plus analyzer (e.g., albumin (ALB), alkaline phosphatase (ALP), alanine aminotransferase (ALT), aspartate aminotransferase (AST), blood urea nitrogen (BUN), etc.) [22]. Electrolyte levels [sodium (Na+), potassium (K+) and chloride (Cl)] were evaluated using a 9180 Electrolyte Analyzer [22]. Serum thyroid hormones [tri‐iodothyronine (T3), thyroxine (T4) and thyroid‐stimulating hormone (TSH)] were measured using ELISA kits from Wuhan Huamei Biotech Co., Ltd. Additionally, urine samples were collected and analysed for various parameters using a Roche Cobas u411 urine analyser [22].

### 2.7. Pathology

At the end of the study, animals were humanely euthanised by CO_2_ asphyxiation followed by exsanguination. A thorough pathological examination was conducted on all animals, involving a detailed gross necropsy. Organs such as the liver, kidneys, adrenals, testes, epididymides, uterus, ovaries, thymus, spleen, lung, brain, eyes, thyroid with parathyroid, pituitary, prostate, plus seminal vesicles with coagulation glands and heart were weighed immediately after collection and before fixation.

All collected organs (excluding eyes and testes) were preserved in a 10% neutral‐buffered formalin solution, while eyes and testes were preserved in modified Davidson’s fluid. Subsequently, histopathological examination was performed on the preserved organs and tissues from both the vehicle control (G1) and high‐dose group (G4) rats. Tissues were processed for routine paraffin embedding, and sections were stained with Mayer’s haematoxylin and eosin stain. Additionally, testes were sectioned at 4 microns and stained with PAS (periodic acid–Schiff) reagent and haematoxylum to aid in the qualitative assessment of spermatogenesis. This ensured a detailed evaluation of any histopathological changes or abnormalities present in the organs and tissues of the study animals.

### 2.8. Statistical Analysis

All the data were checked for normality with the normality test (Kolmogorov–Smirnov (Lilliefors), Shapiro–Wilk W, D’Agostino–Pearson Skewness, Kurtosis and Omnibus K2 tests). Data for each animal group were subjected to analysis of variance (ANOVA). Values were given as mean ± standard deviation (SD). *T*‐test was used to compare the difference between the treated and control groups. The statistical significance of differences was calculated with a one‐way ANOVA. All analyses and comparisons were evaluated at the 5% (*p* ≤ 0.05) level. Statistical analysis was performed using Graphpad Prism Software Inc., CA, USA (Version: 10.0.5).

## 3. Results

### 3.1. In‐Life Observations

Throughout the treatment period, no mortality, clinical signs or functional deficits were observed in any of the animals across the six groups. Additionally, no ocular abnormalities were detected in any animals before or at the end of the treatment period in the vehicle control and high‐dose group animals. Although there was an observed increase in body weight across all groups, no statistically significant changes in mean body weights were noted in the treated groups compared to those in their respective control groups during both the treatment and recovery periods (Table 1).

**TABLE 1:** Initial and terminal body weight (g).

Group		Bodyweight (g)			
		Male		Female	
		Initial week	Terminal week	Initial week	Terminal week
G1	Vehicle control	338.29 ± 17.52	631.3 ± 55.38	209.04 ± 11.98	315.61 ± 39.93
G2	Low‐dose Zylaria	338.28 ± 19.6	608.05 ± 56.46	210.46 ± 15.38	314.15 ± 23.09
G3	Mid‐dose Zylaria	340.27 ± 17.08	605.81 ± 61.57	207.91 ± 18.74	306.00 ± 27.06
G4	High‐dose Zylaria	343.58 ± 15.99	624.93 ± 42.05	208.67 ± 16.84	313.63 ± 26.33
G1R	Vehicle control recovery	353.14 ± 8.91	658.20 ± 20.93	210.84 ± 10.21	311.46 ± 13.80
G4R	High dose of Zylaria recovery	351.81 ± 14.10	627.78 ± 42.65	213.08 ± 14.21	319.40 ± 24.78

*Note:* Values as mean ± SD (main groups, *n* = 10 rats each and recovery groups, *n* = 5 rats each).

Weekly measurements of feed consumption revealed a statistically significant decrease in feed consumption in males on days 22–29 and days 29–36 in the G3 main group compared to that of the control group. However, in the female main group and both the male and female recovery groups, no statistically significant changes in feed consumption were observed compared to their respective control groups [Table S4]. These changes in feed consumption were inconsistent throughout the treatment and, hence, were not considered treatment‐related.

### 3.2. Clinical Pathology

#### 3.2.1. Haematology

Compared with the control group, all doses of Zylaria demonstrated no significant effect on any of the haematological parameters in both male and female animals. However, in males, a statistically significant decrease in the reticulocyte count in G2 compared to that of the vehicle control group and an increase in the relative monocyte count in G4R compared to that in G1R were observed. On the other hand, in females, a statistically significant decrease in MCV in G2 compared to that in the vehicle control group and an increase in MCHC in G3 and G4 compared with that in the vehicle control group were observed (Table 2).

**TABLE 2:** Haematological findings.

Group		G1	G2	G3	G4	G1R	G4R
Parameters	Sex	Vehicle control	Low‐dose zylaria	Mid‐dose zylaria	High‐dose zylaria	Vehicle control recovery	High dose of zylaria recovery
RBC (10^6^/µL)	Male	8.94 ± 0.45	8.88 ± 0.52	8.76 ± 0.73	9.16 ± 0.46	8.99 ± 0.35	8.96 ± 0.45
	Female	7.68 ± 0.50	8.11 ± 0.39	7.70 ± 0.38	7.67 ± 0.37	8.79 ± 0.81	8.54 ± 0.64

HGB (g/dL)	Male	16.46 ± 0.75	16.13 ± 0.93	16.23 ± 0.95	16.39 ± 0.68	16.14 ± 0.32	16.28 ± 0.42
	Female	15.07 ± 0.80	15.81 ± 0.69	15.16 ± 0.77	15.19 ± 0.61	15.94 ± 0.86	16.06 ± 0.27

HCT (%)	Male	48.26 ± 2.05	47.04 ± 2.49	47.42 ± 2.78	47.80 ± 1.67	47.24 ± 1.27	47.80 ± 0.79
	Female	43.69 ± 2.61	44.73 ± 2.24	42.73 ± 1.97	42.75 ± 1.82	46.78 ± 2.93	46.58 ± 1.44

MCV(µ·m^3^)	Male	54.00 ± 1.43	53.02 ± 1.16	54.30 ± 2.29	52.22 ± 2.28	52.62 ± 1.68	53.48 ± 2.64
	Female	56.99 ± 3.00	55.18^a^ ± 0.96	55.54 ± 1.63	55.75 ± 0.94	53.38 ± 1.86	54.70 ± 2.82

MCH (pg)	Male	18.44 ± 0.47	18.18 ± 0.43	18.60 ± 0.69	17.92 ± 0.77	17.98 ± 0.79	18.18 ± 0.95
	Female	19.64 ± 0.52	19.51 ± 0.46	19.71 ± 0.67	19.80 ± 0.27	18.22 ± 0.88	18.84 ± 1.16

MCHC (g/dL)	Male	34.14 ± 0.40	34.28 ± 0.33	34.26 ± 0.51	34.33 ± 0.72	34.22 ± 0.61	34.00 ± 0.41
	Female	34.51 ± 1.14	35.35 ± 0.55	35.49^a^ ± 0.41	35.53^a^ ± 0.58	34.10 ± 0.73	34.46 ± 0.61

RDW (%)	Male	13.21 ± 0.61	13.26 ± 0.40	13.29 ± 0.71	13.27 ± 0.43	14.92 ± 1.04	14.42 ± 0.49
	Female	13.94 ± 2.6	12.90 ± 0.9	12.72 ± 0.5	12.53 ± 0.6	14.70 ± 0.7	14.26 ± 1.0

PLT (10^3^/µL))	Male	986.20 ± 176.66	851.60 ± 158.38	952.30 ± 121.60	932.50 ± 203.01	1059.80 ± 50.69	1055.20 ± 144.35
	Female	914.80 ± 175.92	827.50 ± 83.88	859.00 ± 168.49	851.10 ± 82.23	1073.60 ± 191.46	956.20 ± 204.42

MPV (µm^3^)	Male	9.85 ± 0.41	9.90 ± 0.37	9.92 ± 0.29	9.86 ± 0.38	9.70 ± 0.29	9.58 ± 0.47
	Female	9.66 ± 0.36	9.73 ± 0.25	9.91 ± 0.40	9.89 ± 0.39	9.50 ± 0.61	9.72 ± 0.54

Retic (%)	Male	3.24 ± 0.50	2.63^a^ ± 0.36	2.75 ± 0.21	2.81 ± 0.18	2.34 ± 0.13	2.20 ± 0.27
	Female	2.40 ± 0.16	2.41 ± 0.22	2.51 ± 0.18	2.38 ± 0.17	2.26 ± 0.09	2.26 ± 0.11

WBC (10^3^/µL)	Male	11.72 ± 2.00	11.31 ± 1.46	11.12 ± 2.21	11.18 ± 2.40	10.59 ± 2.95	9.63 ± 1.36
	Female	9.22 ± 2.71	8.39 ± 1.58	8.01 ± 1.90	9.05 ± 2.76	8.36 ± 1.81	8.10 ± 2.36

NEU (%)	Male	33.81 ± 9.55	30.10 ± 6.10	28.32 ± 7.02	32.62 ± 12.30	22.66 ± 7.46	27.54 ± 7.30
	Female	23.87 ± 6.62	31.18 ± 16.66	29.24 ± 9.43	33.89 ± 18.38	26.26 ± 6.95	22.96 ± 4.76

LYM (%)	Male	55.87 ± 10.71	58.46 ± 8.14	62.40 ± 7.66	56.32 ± 12.03	70.52 ± 9.32	64.18 ± 7.17
	Female	69.14 ± 7.14	61.40 ± 17.08	62.02 ± 10.27	58.98 ± 18.66	65.66 ± 8.99	69.00 ± 5.89

MON (%)	Male	8.44 ± 2.39	9.58 ± 2.63	7.54 ± 2.08	9.16 ± 1.11	4.48 ± 1.09	6.06^a^ ± 0.70
	Female	4.78 ± 1.66	4.99 ± 1.54	6.43 ± 3.19	5.01 ± 1.12	5.82 ± 2.04	5.96 ± 1.25

EOS (%)	Male	1.88 ± 0.39	1.86 ± 0.33	1.74 ± 0.25	1.90 ± 0.36	2.34 ± 0.91	2.22 ± 0.63
	Female	2.21 ± 0.49	2.43 ± 0.59	2.31 ± 0.53	2.12 ± 0.30	2.26 ± 0.65	2.08 ± 0.59

BAS (%)	Male	0.00 ± 0.00	0.00 ± 0.00	0.00 ± 0.00	0.00 ± 0.00	0.00 ± 0.00	0.00 ± 0.00
	Female	0.00 ± 0.00	0.00 ± 0.00	0.00 ± 0.00	0.00 ± 0.00	0.00 ± 0.00	0.00 ± 0.00

*Note:* Values as mean ± SD (main groups treated for 90 days, *n* = 10 each; recovery groups, *n* = 5).

^a^Statistically significant change at *p* < 0.05 compared to respective vehicle control and recovery control vehicle group.

#### 3.2.2. Coagulation Parameter

In males, a statistically significant increase in PT was observed in G4 compared to that of the vehicle control group. Conversely, in females, a statistically significant increase in APTT was noted in G4R compared to that in the G1R. Interestingly, the changes observed in coagulation parameters could not be correlated with any changes in related parameters, suggesting potential independent effects on coagulation profiles [Table S5].

#### 3.2.3. Clinical Chemistry

The clinical chemistry analysis revealed noteworthy findings (Table 3). Among male rats administered a mid‐dose of Zylaria (G3), there was a statistically significant increase in glucose levels and a decrease in triglycerides compared to the vehicle group. Furthermore, an increase in the A/G ratio was observed in G4 compared to that in the vehicle control group (G1), alongside a decrease in ALP in G4R compared to that in G1R. Conversely, in females, significant differences were noted, including a decrease in AST in the G4 group, an increase in total bilirubin in G3 and decreases in calcium and potassium in G2, G3 and G4 compared to G1. Additionally, increases in urea, creatinine, inorganic phosphorus and BUN, along with a decrease in ALP, were observed in G4R compared to that in G1R. The observed changes in clinical chemistry parameters were sporadic, lacked dose dependency, were not consistent across sexes and were not associated with any correlated gross or histopathological findings; therefore, these changes were considered toxicologically irrelevant and unrelated to the test item.

**TABLE 3:** Clinical chemistry data.

Group		G1	G2	G3	G4	G1R
Parameters Sex	Sex	Vehicle control	Low‐dose zylaria	Mid‐dose zylaria	High‐dose zylaria	Vehicle control recovery
GLU (mg/dL)	Male	114.35 ± 15.80	109.62 ± 19.39	135.21^a^ ± 25.66	123.00 ± 12.73	114.54 ± 21.68
	Female	123.75 ± 21.69	109.98 ± 19.10	117.34 ± 17.18	106.93 ± 6.80	115.31 ± 70.89

UREA (mg/dL)	Male	25.58 ± 3.10	27.63 ± 3.92	28.50 ± 3.84	27.37 ± 1.73	28.19 ± 4.40
	Female	34.10 ± 6.05	36.76 ± 6.47	35.42 ± 3.10	32.81 ± 4.30	33.70 ± 1.90

CRE (mg/dL)	Male	0.70 ± 0.04	0.73 ± 0.07	0.74 ± 0.05	0.72 ± 0.05	0.90 ± 0.02
	Female	0.81 ± 0.07	0.79 ± 0.07	0.79 ± 0.03	0.78 ± 0.04	1.04 ± 0.10

TCHO (mg/dL)	Male	69.24 ± 15.30	60.38 ± 14.84	60.45 ± 11.91	67.87 ± 13.68	62.01 ± 3.00
	Female	87.48 ± 150.74	82.58 ± 25.03	86.61 ± 21.34	84.36 ± 29.88	67.94 ± 12.48

TRIG (mg/dL)	Male	80.31 ± 23.36	73.86 ± 25.73	56.27^a^ ± 21.27	69.60 ± 15.97	93.28 ± 25.83
	Female	57.07 ± 12.88	49.55 ± 9.66	53.16 ± 10.48	48.68 ± 9.72	49.73 ± 8.55

AST (U/L)	Male	192.27 ± 37.91	196.69 ± 32.16	166.49 ± 2127	167.88 ± 29.39	176.26 ± 24.41
	Female	159.17 ± 19.52	149.10 ± 23.99	147.67 ± 22.89	129.74^a^ ± 21.24	124.43 ± 33.87

ALT (U/L)	Male	65.71 ± 11.22	70.55 ± 15.66	68.34 ± 15.23	64.68 ± 9.86	56.56 ± 14.91
	Female	48.87 ± 15.02	56.44 ± 16.23	55.68 ± 16.01	49.92 ± 10.12	44.28 ± 22.55

TP (g/L)	Male	76.60 ± 1.59	76.16 ± 5.12	75.91 ± 2.47	76.77 ± 3.13	65.17 ± 1.59
	Female	83.83 ± 5.83	80.76 ± 6.06	81.83 ± 6.08	81.84 ± 2.71	71.54 ± 2.85

HDL (mg/dL)	Male	22.65 ± 2.55	20.46 ± 3.53	20.59 ± 2.98	21.41 ± 2.83	20.93 ± 1.46
	Female	40.22 ± 4.63	34.95 ± 7.64	37.48 ± 7.26	36.13 ± 9.06	30.72 ± 5.07

LDL (mg/dL)	Male	9.12 ± 2.52	8.51 ± 2.19	7.67 ± 2.68	8.32 ± 2.35	7.74 ± 1.38
	Female	7.28 ± 1.99	5.87 ± 1.42	6.22 ± 1.50	7.58 ± 3.97	5.34 ± 1.04

ALB (g/L)	Male	43.26 ± 0.83	43.08 ± 2.72	43.32 ± 1.23	44.48 ± 1.24	37.52 ± 0.47
	Female	48.36 ± 4.42	47.31 ± 3.10	48.51 ± 3.51	47.89 ± 2.08	43.42 ± 1.40

T.BIL (mg/dL)	Male	0.23 ± 0.11	0.23 ± 0.08	0.22 ± 0.09	0.16 ± 0.09	0.15 ± 0.05
	Female	0.15 ± 0.06	0.20 ± 0.04	0.26^a^ ± 0.08	0.22 ± 0.08	0.16 ± 0.03

Ca (mg/dL)	Male	10.72 ± 0.81	10.14 ± 0.67	10.56 ± 0.70	10.85 ± 0.97	9.42 ± 0.37
	Female	11.56 ± 1.16	10.21^a^ ± 0.55	10.18^a^ ± 0.73	10.03^a^ ± 0.54	9.65 ± 0.54

PHO (mg/dL)	Male	8.07 ± 1.00	8.04 ± 0.93	8.21 ± 0.87	8.63 ± 1.53	6.76 ± 0.55
	Female	6.90 ± 1.12	6.91^a^ ± 0.72	6.74^a^ ± 0.37	6.20^a^ ± 0.48	3.94 ± 1.12

GLOB (g/L)	Male	33.34 ± 1.15	33.09 ± 2.85	32.60 ± 1.31	32.30 ± 2.21	27.65 ± 1.42
	Female	35.47 ± 2.70	33.44 ± 3.37	33.33 ± 3.00	33.96 ± 1.38	28.12 ± 1.66

TBA (µmol/L)	Male	45.11 ± 41.83	43.49 ± 25.50	64.21 ± 49.27	27.68 ± 16.89	30.14 ± 24.49
	Female	50.94 ± 48.22	79.38 ± 67.87	39.54 ± 45.84	55.13 ± 38.89	40.73 ± 34.04

ALP (U/L)	Male	71.11 ± 10.15	72.10 ± 15.64	73.99 ± 22.19	68.49 ± 6.20	90.70 ± 22.87
	Female	50.39 ± 18.47	36.41 ± 14.17	44.33 ± 13.34	43.80 ± 14.01	50.18 ± 7.94

BUN (mg/dL)	Male	11.96 ± 1.45	12.91 ± 1.83	13.32 ± 1.79	12.79 ± 0.81	13.17 ± 2.06
	Female	15.93 ± 2.83	17.18 ± 3.02	16.55 ± 1.45	15.33 ± 2.01	15.75 ± 0.89

A/G Ratio	Male	1.30 ± 0.05	1.31 ± 0.08	1.33 ± 0.03	1.38^a^ ± 0.08	1.36 ± 0.07
	Female	1.36 ± 0.10	1.42 ± 0.09	1.46 ± 0.09	1.41 ± 0.07	1.55 ± 0.07

NA (mmol/L)	Male	141.50 ± 2.46	142.10 ± 1.10	142.30 ± 2.11	141.30 ± 1.83	141.40 ± 1.14
	Female	142.80 ± 1.69	141.20 ± 1.23	141.30 ± 1.16	141.30 ± 1.16	142.20 ± 0.45

K (mmol/L)	Male	5.33 ± 0.25	4.99 ± 0.39	5.24 ± 0.45	5.10 ± 0.39	5.10 ± 0.32
	Female	5.06 ± 0.46	4.52^a^ ± 0.18	4.57^a^ ± 0.37	4.60^a^ ± 0.29	4.84 ± 0.55

Cl (mmol/L)	Male	100.60 ± 2.37	101.50 ± 1.18	101.80 ± 1.40	100.80 ± 0.39	101.40 ± 1.52
	Female	102.10 ± 1.97	102.30 ± 1.49	101.60 ± 0.84	103.10 ± 2.13	103.80 ± 1.64

*Note:* Values as mean ± SD (main groups treated for 90 days, *n* = 10 each; recovery groups, *n* = 5).

^a^Statistically significant change at *p* < 0.05 in comparison to the respective vehicle control and recovery control vehicle group.

#### 3.2.4. Thyroid Parameter

A statistically significant increase in T4 levels was noted in the G2 and G3 groups compared to that in the corresponding vehicle control group. However, no statistically significant difference was observed in all other thyroid hormone levels across the Zylaria‐treated groups. Mainly, no significant changes in absolute or relative thyroid weight were observed, and all thyroids with parathyroid examined in the study were normal upon gross and histopathological examinations. The changes in thyroid hormone levels seen in both male and female rats were considered toxicologically irrelevant (Table 4).

**TABLE 4:** Thyroid parameters.

Group		G1	G2	G3	G4	G1R	G4R
Parameters	Sex	Vehicle control	Low‐dose zylaria	Mid‐dose zylaria	High‐dose zylaria	Vehicle control recovery	High dose of zylaria recovery
T3 (ng/mL)	Male	1.982 ± 0.043	2.001 ± 0.043	1.973 ± 0.089	1.926 ± 0.088	1.534 ± 0.194	1.545 ± 0.205
T4 (ng/mL)		79.532 ± 0.806	80.735^a^ ± 0.404	80.303^a^ ± 0.782	79.668 ± 0.687	80.030 ± 1.226	80.514 ± 0.677
TSH (pg/mL)		628.465 ± 50.511	616.311 ± 55.802	641.959 ± 72.275	662.720 ± 69.061	887.188 ± 150.353	871.449 ± 85.304
T3 (ng/mL)	Female	1.690 ± 0.274	1.707 ± 0.172	1.537 ± 0.287	1.442 ± 0.312	1.739 ± 0.245	1.576 ± 0.233
T4 (ng/mL)		79.293 ± 0.909	79.194 ± 1.684	79.318 ± 1.429	79.955 ± 1.545	79.145 ± 0.7	79.585 ± 1.258
TSH (pg/mL)		808.719 ± 191.766	783.832 ± 137.613	838.122 ± 107.434	957.915 ± 235.225	790.062 ± 155.995	880.978 ± 170.645

*Note:* Values are mean ± SD for main groups (*n* = 10) and recovery groups (*n* = 5).

^a^Statistically significant change at *p* < 0.05 when compared with the vehicle control group.

#### 3.2.5. Urinalysis

No significant treatment‐emergent adverse effects and changes were observed in any treatment group, and the results were comparable to those of the control groups. However, in males, a statistically significant change in specific gravity was noted in group G2, while in females, a statistically significant decrease in urine volume was observed in group G4 compared to that of the vehicle control group. Thus, the changes were considered normal biological variations and did not have toxicological significance [Table S6a–S6b].

### 3.3. Pathology

#### 3.3.1. Necropsy and Gross Pathology

Gross observations revealed bilateral small‐sized testes and epididymides in one male rat and unilateral small‐sized testes in another male rat of the G4 group [Table S7]. These findings were observed in individual animals only, lacked dose dependency and are recognised as spontaneous background findings in laboratory rats; in the absence of consistent histopathological correlates, they were considered toxicologically irrelevant and unrelated to the test item.

#### 3.3.2. Organ Weights

In females, no treatment‐emergent adverse effect was observed in either the absolute or relative organ weights. However, in male animals, statistically significant changes in both absolute and relative pituitary gland weight were observed in G3 and G4 compared to those in the G1 group (Tables 5 and 6). It is noteworthy that the observed changes in the pituitary gland were limited to the single‐sex group, with no gross or histopathological changes noted in the G4 group. Therefore, these changes were considered unrelated to the test item.

**TABLE 5:** Absolute organ weight (g).

Group		G1	G2	G3	G4	G1R	G4R
Organ	Sex	Vehicle control	Low‐dose zylaria	Mid‐dose zylaria	High‐dose zylaria	Vehicle control recovery	High dose of zylaria recovery
Liver	Male	19.983 ± 2.202	19.340 ± 2.408	19.617 ± 3.048	20.546 ± 2.165	19.552 ± 0.777	20.097 ± 2.784
	Female	10.571 ± 1.111	10.038 ± 1.348	10.276 ± 0.789	10.584 ± 0.805	9.459 ± 0.680	9.820 ± 0.893

Kidneys	Male	4.128 ± 0.583	4.324 ± 0.399	4.142 ± 0.461	4.250 ± 0.541	4.157 ± 0.201	4.387 ± 0.729
	Female	2.245 ± 0.290	2.257 ± 0.221	2.272 ± 0.278	2.208 ± 0.185	2.192 ± 0.248	2.230 ± 0.358

Spleen	Male	0.927 ± 0.144	1.031 ± 0.115	0.956 ± 0.158	0.999 ± 0.114	0.990 ± 0.139	1.073 ± 0.099
	Female	0.730 ± 0.109	0.703 ± 0.118	0.649 ± 0.032	0.688 ± 0.052	0.534 ± 0.042	0.565 ± 0.047

Adrenals	Male	0.091 ± 0.012	0.095 ± 0.010	0.093 ± 0.010	0.098 ± 0.009	0.096 ± 0.004	0.096 ± 0.010
	Female	0.114 ± 0.008	0.111 ± 0.008	0.117 ± 0.009	0.108 ± 0.005	0.104 ± 0.010	0.101 ± 0.009

Heart	Male	2.091 ± 0.460	2.029 ± 0.210	1.959 ± 0.197	2.060 ± 0.201	2.191 ± 0.152	2.106 ± 0.242
	Female	1.277 ± 0.163	1.179 ± 0.126	1.162 ± 0.115	1.204 ± 0.150	1.268 ± 0.192	1.147 ± 0.143

Thymus	Male	0.513 ± 0.084	0.538 ± 0.084	0.544 ± 0.085	0.506 ± 0.049	0.527 ± 0.046	0.562 ± 0.114
	Female	0.416 ± 0.081	0.402 ± 0.051	0.401 ± 0.049	0.399 ± 0.034	0.387 ± 0.032	0.360 ± 0.039

Brain	Male	2.262 ± 0.135	2.354 ± 0.190	2.306 ± 0.192	2.329 ± 0.187	2.294 ± 0.099	2.320 ± 0.136
	Female	2.078 ± 0.142	2.065 ± 0.096	2.093 ± 0.092	2.096 ± 0.154	2.119 ± 0.179	2.094 ± 0.134

Testes	Male	3.714 ± 0.334	3.943 ± 0.406	3.864 ± 0.335	3.400 ± 0.871	3.708 ± 0.374	4.169 ± 0.400
Ovaries	Female	0.243 ± 0.027	0.235 ± 0.025	0.238 ± 0.031	0.240 ± 0.032	0.219 ± 0.021	0.235 ± 0.014
Epididymides	Male	1.797 ± 0.153	1.887 ± 0.426	1.751 ± 0.192	1.629 ± 0.293	1.690 ± 0.191	1.805 ± 0.359
Uterus	Female	0.855 ± 0.125	0.800 ± 0.093	0.822 ± 0.094	0.806 ± 0.091	0.792 ± 0.115	0.750 ± 0.161
Thyroid with Parathyroid	Male	0.039 ± 0.005	0.039 ± 0.003	0.040 ± 0.002	0.042 ± 0.003	0.039 ± 0.003	0.039 ± 0.003
	Female	0.039 ± 0.003	0.041 ± 0.003	0.039 ± 0.005	0.038 ± 0.003	0.038 ± 0.003	0.039 ± 0.002

Pituitary gland	Male	0.021 ± 0.002	0.022 ± 0.003	0.024^a^ ± 0.003	0.023^a^ ± 0.002	0.028 ± 0.004	0.028 ± 0.002
	Female	0.024 ± 0.003	0.022 ± 0.002	0.023 ± 0.002	0.022 ± 0.003	0.032 ± 0.003	0.030 ± 0.002

Prostate + seminal vesicles with coagulating glands	Male	4.463 ± 0.511	4.633 ± 0.376	4.606 ± 0.794	4.519 ± 0.639	4.619 ± 0.691	4.735 ± 0.345

*Note:* Values are mean ± SD for the main (*n* = 10) treated for 90 days and recovery (*n* = 5) groups.

^a^Statistically significant change at *p* < 0.05 when compared with the vehicle control group.

**TABLE 6:** Relative organ weights (%).

Group		G1	G2	G3	G4	G1R	G4R
Organ	Sex	Vehicle control	Low‐dose zylaria	Mid‐dose zylaria	High‐dose zylaria	Vehicle control recovery	High‐zylaria recovery
Liver	Male	3.333 ± 0.370	3.334 ± 0.292	3.394 ± 0.268	3.451 ± 0.293	3.109 ± 0.183	3.354 ± 0.283
	Female	3.572 ± 0.265	3.350 ± 0.367	3.560 ± 0.302	3.600 ± 0.198	3.217 ± 0.209	3.229 ± 0.160

Kidneys	Male	0.689 ± 0.060	0.746 ± 0.048	0.721 ± 0.071	0.715 ± 0.078	0.660 ± 0.031	0.731 ± 0.074
	Female	0.758 ± 0.070	0.756 ± 0.072	0.784 ± 0.063	0.753 ± 0.072	0.747 ± 0.089	0.732 ± 0.088

Spleen	Male	0.155 ± 0.020	0.179 ± 0.023	0.167 ± 0.029	0.167 ± 0.014	0.157 ± 0.022	0.180 ± 0.018
	Female	0.247 ± 0.030	0.236 ± 0.046	0.226 ± 0.021	0.235 ± 0.023	0.182 ± 0.016	0.187 ± 0.025

Adrenals	Male	0.015 ± 0.001	0.017 ± 0.002	0.016 ± 0.002	0.017 ± 0.002	0.015 ± 0.001	0.016 ± 0.002
	Female	0.039 ± 0.005	0.037 ± 0.004	0.040 ± 0.004	0.037 ± 0.003	0.036 ± 0.005	0.033 ± 0.003

Heart	Male	0.348 ± 0.062	0.351 ± 0.038	0.342 ± 0.038	0.346 ± 0.027	0.348 ± 0.029	0.352 ± 0.026
	Female	0.432 ± 0.051	0.393 ± 0.020	0.402 ± 0.035	0.411 ± 0.056	0.432 ± 0.068	0.377 ± 0.039

Thymus	Male	0.086 ± 0.011	0.093 ± 0.011	0.095 ± 0.014	0.085 ± 0.008	0.084 ± 0.007	0.095 ± 0.023
	Female	0.140 ± 0.024	0.135 ± 0.018	0.140 ± 0.023	0.136 ± 0.013	0.132 ± 0.008	0.119 ± 0.014

Brain	Male	0.380 ± 0.026	0.408 ± 0.040	0.403 ± 0.051	0.392 ± 0.036	0.365 ± 0.019	0.390 ± 0.040
	Female	0.710 ± 0.107	0.692 ± 0.047	0.726 ± 0.052	0.715 ± 0.073	0.722 ± 0.074	0.690 ± 0.046
Testes	Male	0.626 ± 0.079	0.681 ± 0.064	0.677 ± 0.092	0.573 ± 0.148	0.590 ± 0.069	0.701 ± 0.088
Ovaries	Female	0.082 ± 0.011	0.079 ± 0.011	0.083 ± 0.012	0.082 ± 0.015	0.075 ± 0.009	0.077 ± 0.007
Epididymides	Male	0.302 ± 0.030	0.328 ± 0.081	0.307 ± 0.049	0.275 ± 0.053	0.269 ± 0.032	0.301 ± 0.056
Uterus	Female	0.291 ± 0.051	0.269 ± 0.044	0.286 ± 0.040	0.275 ± 0.041	0.269 ± 0.035	0.249 ± 0.062
Thyroid with parathyroid	Male	0.006 ± 0.001	0.007 ± 0.001	0.007 ± 0.001	0.007 ± 0.001	0.006 ± 0.000	0.007 ± 0.001
	Female	0.013 ± 0.002	0.014 ± 0.002	0.014 ± 0.002	0.013 ± 0.002	0.013 ± 0.001	0.013 ± 0.001

Pituitary gland	Male	0.003 ± 0.001	0.004 ± 0.001	0.004^a^ ± 0.001	0.004^a^ ± 0.001	0.004 ± 0.001	0.005 ± 0.001
	Female	0.008 ± 0.002	0.008 ± 0.001	0.008 ± 0.001	0.008 ± 0.001	0.011 ± 0.001	0.010 ± 0.001

Prostate + seminal vesicles with coagulating glands	Male	0.751 ± 0.107	0.806 ± 0.110	0.804 ± 0.149	0.759 ± 0.099	0.734 ± 0.108	0.796 ± 0.094

*Note:* Values are mean ± SD for the main (*n* = 10) treated for 90 days and recovery (*n* = 5) groups.

^a^Statistically significant change at *p* < 0.05 when compared with the vehicle control group.

#### 3.3.3. Histopathology

Pathological examination revealed no observable lesions in any of the excised organs in either male or female rats receiving repeated different doses of Zylaria. However, certain animals in both the control and Zylaria‐treated groups exhibited slightly abnormal histological appearances in organ anatomy. For example, in the liver of the G1 group, hepatocellular vacuolation (focal and minimal in severity) was observed in one male animal, along with hepatocellular necrosis (focal and minimal in severity) in another male animal. Additionally, in the kidney of the G1 group, one male animal exhibited the presence of a cyst in the medulla (unilateral) and dilated pelvis (unilateral) in another male animal. Furthermore, chronic inflammatory foci (multifocal and minimal in severity) were detected in the lungs of two G1 males and one G1 female.

In contrast, vacuolation (multifocal and minimal in severity) of the zona fasciculata of the adrenal glands (bilateral) was observed in one male of both the G1 and G4 groups, as well as one female of the G1 group. Other findings included unilateral atrophy/degeneration of seminiferous tubules (diffuse and moderate in severity) in one G4 male, bilateral atrophy of seminiferous tubules (diffuse and severe in severity) in one G4 male, bilateral aspermia in the epididymides of one male from the G4 group, unilateral oligospermia in the epididymides of another male from the G4 group and the presence of a follicular cyst in one female animal of the G1 group (Figure 1). The microscopic findings observed in the testes and epididymides were identified in the same individual animals that exhibited the corresponding gross pathological findings at necropsy. These correlated gross and microscopic findings were sporadic, lacked dose dependency and were not accompanied by changes in reproductive organ weights; therefore, they were considered incidental background changes and not toxicologically relevant.

FIGURE 1Histopathological image. Note: (a) G1‐003: adrenal gland—vacuolation, zona fasciculata, 200X; (b) G4‐064: adrenal gland—vacuolation, zona fasciculata, 200X; (c) G1‐002: adrenal gland—normal, 100X; (d) G1‐008: kidneys—dilated pelvis, 100X; (e) G4‐070: kidneys—cyst, medulla, unilateral, 100X; (f) G1‐002: kidneys—normal, 100X; (g) G1‐010: lungs—inflammatory foci, chronic, 100X; (h) G4‐075: lungs—inflammatory foci, chronic, 100X; (i) G1‐002: lungs—normal, 100X; (j) G1‐006: liver—necrosis, hepatocellular, 100X; (k) G1‐004: liver—normal, 100X; (l) G4‐061: testes—atrophy/degeneration, seminiferous tubule, 100X; (m) G1‐006: testes—normal, 100X; (n) G4‐061: epididymides, oligospermia, 100X; (o) G1‐002: epididymides—normal, 100X; (p) G1‐013: ovary—cyst, follicular, 100X and (q) G1‐015: ovary—normal, 100X.

**Figure:**
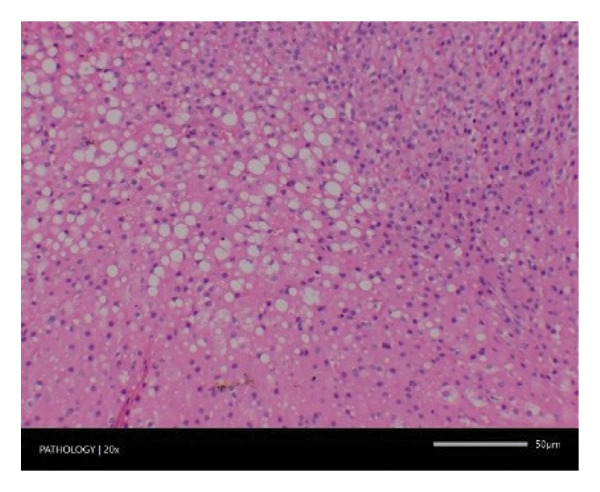
(a)

**Figure:**
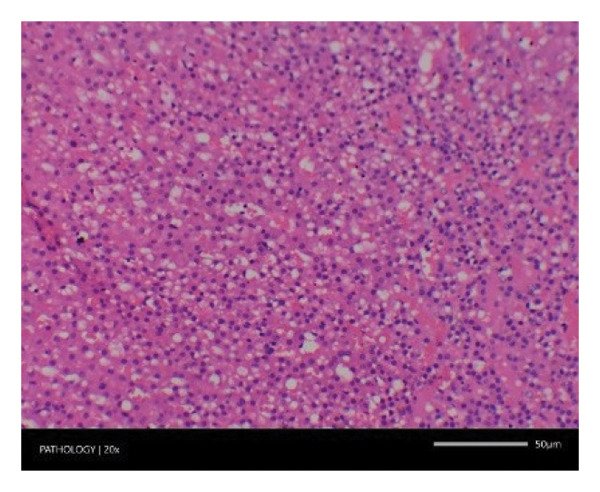
(b)

**Figure:**
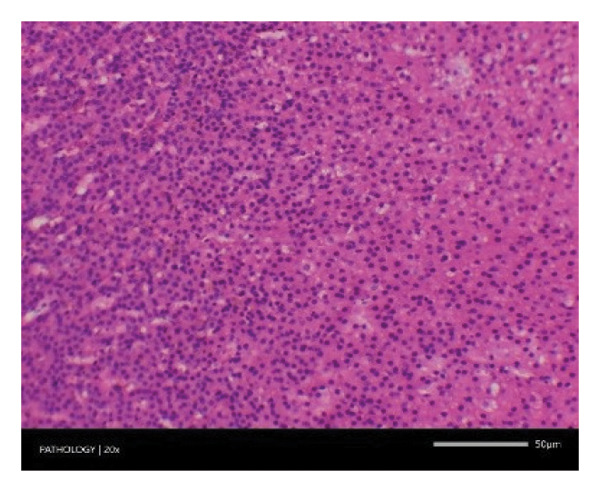
(c)

**Figure:**
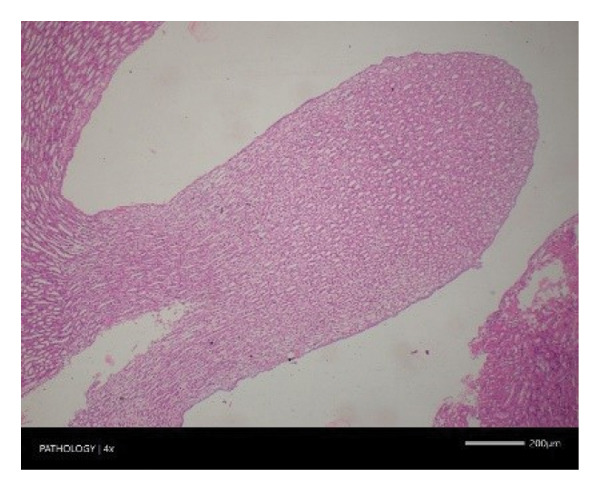
(d)

**Figure:**
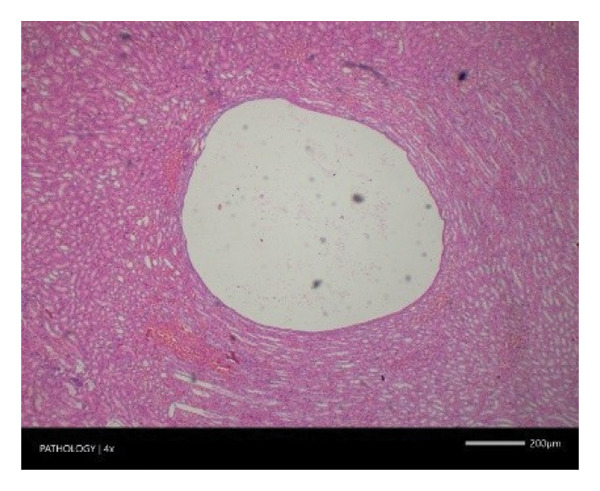
(e)

**Figure:**
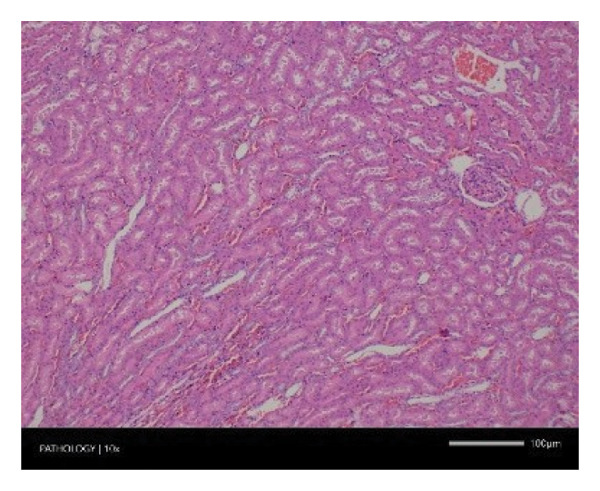
(f)

**Figure:**
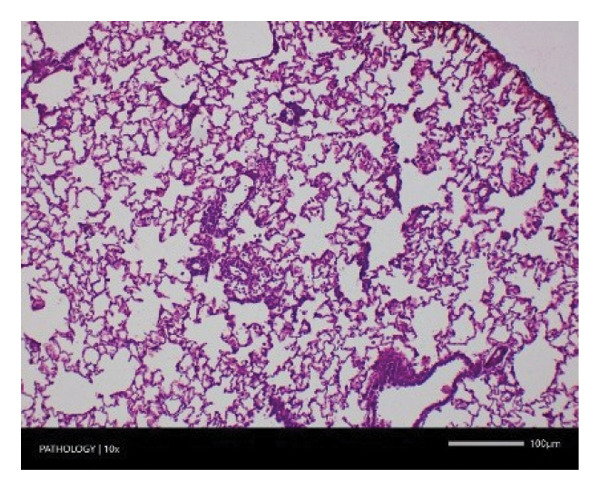
(g)

**Figure:**
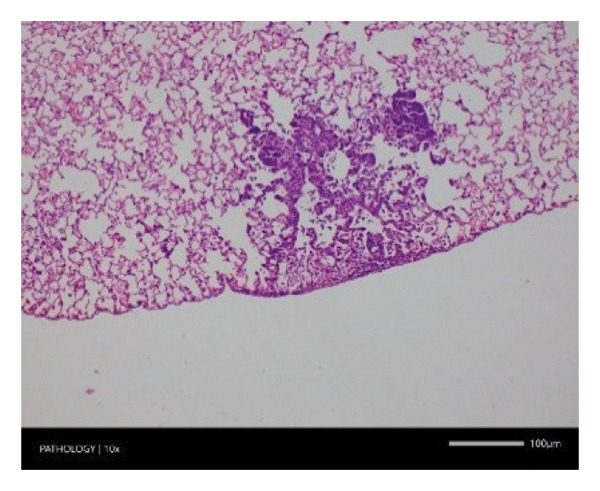
(h)

**Figure:**
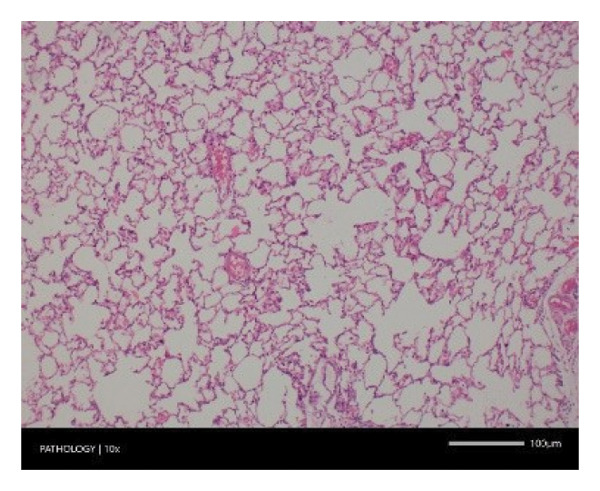
(i)

**Figure:**
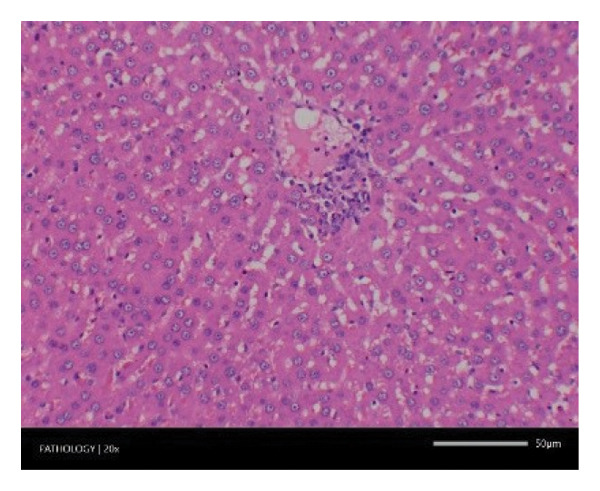
(j)

**Figure:**
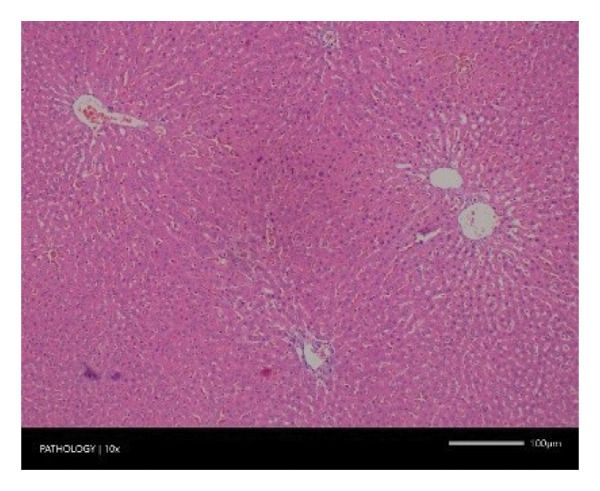
(k)

**Figure:**
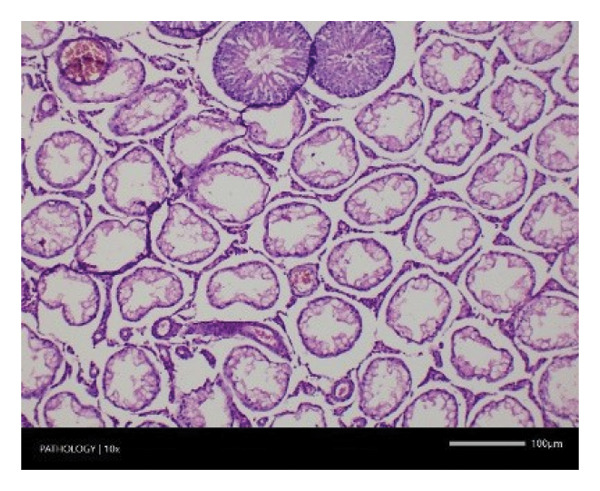
(l)

**Figure:**
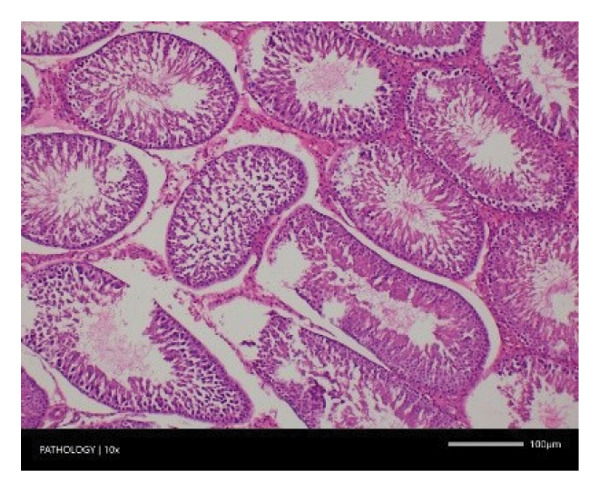
(m)

**Figure:**
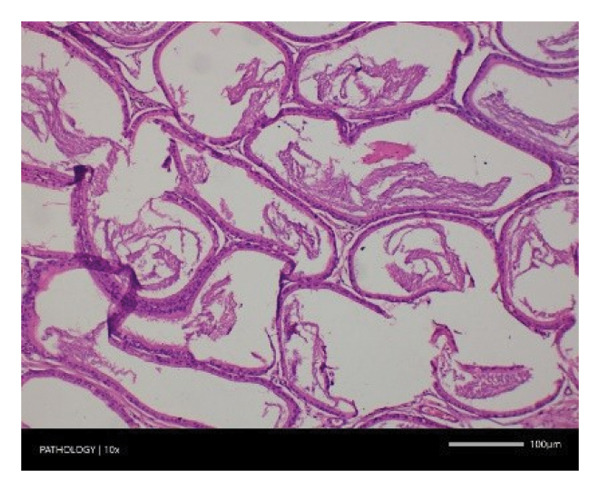
(n)

**Figure:**
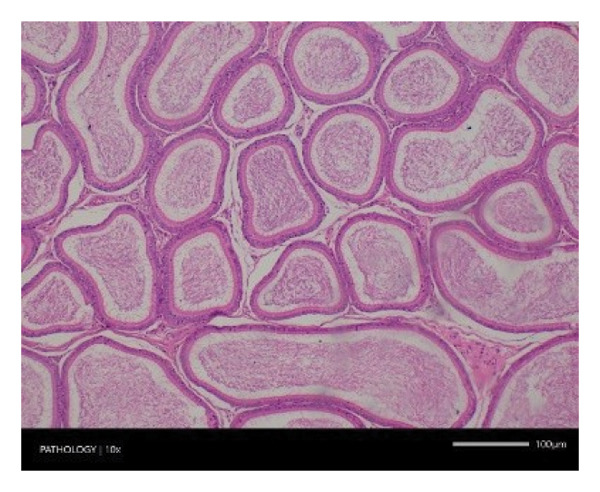
(o)

**Figure:**
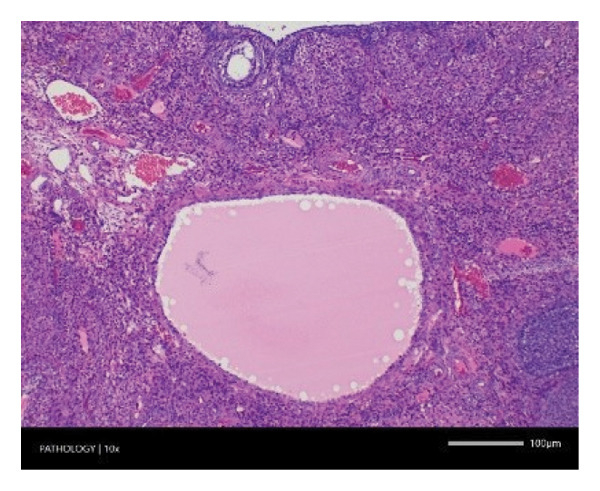
(p)

**Figure:**
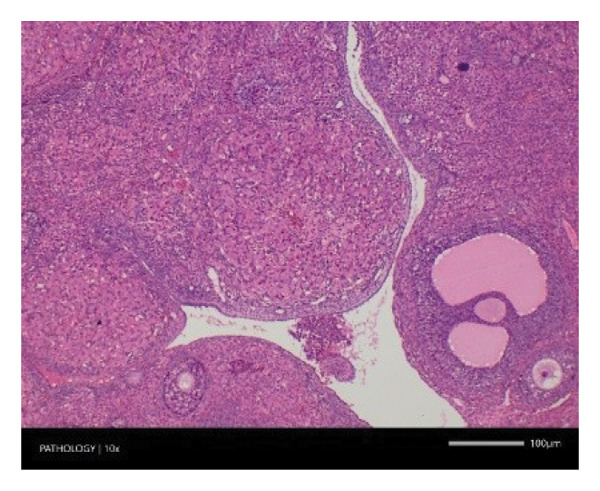
(q)

## 4. Discussion

In the 90‐day repeated dose toxicity study in rats given the Zylaria extract orally at doses of 0 (G1 and G1R), 1000 (G2), 2750 (G3) and 4500 (G4 and G4R) mg/kg bw/day, there was no mortality, change in clinical signs, functional deficits and ocular abnormalities noted. Body weight measurement in experimental animals remained a critical index used to reflect the general health status of animals [25]. In this study, all groups showed an increase in body weight; however, no significant changes were observed. On the other hand, feed consumption in all Zylaria‐treated animals was normal, this indicates that administration of Zylaria at the tested doses did not result in adverse effects on body weight gain or feed consumption when compared with controls. In a 90‐day repeated‐dose oral toxicity study conducted in rats according to the OECD Test Guideline 408, the test item was administered at doses of 1000, 2750 and 4500 mg/kg body weight/day. The NOAEL was established at 4500 mg/kg bw/day, the highest dose tested, with no adverse effects observed. Applying a default uncertainty factor of 100 (10 for interspecies extrapolation and 10 for intraspecies variability), a human acceptable daily intake (ADI) of 45 mg/kg bw/day was calculated. This corresponds to a maximum daily intake of approximately 2700 mg/day for a 60‐kg adult. This results in a margin of exposure of approximately 100 between the highest tested dose in rats and the intended adult human intake, supporting the safe use of the test item as a dietary supplement for healthy adult population.

Toxic compounds primarily target the haematopoietic system, making it a crucial indicator of human and animal health [26]. When compared to control groups, female rats exhibited a statistically significant difference in MCV and MCHC. In contrast, male rats treated with Zylaria showed no significant effects on parameters such as RBC count, haemoglobin levels, HCT and MCH values. This suggests that Zylaria does not affect erythropoiesis, RBC morphology or osmotic fragility in male rats. Furthermore, in both male and female rats, insignificant variations were observed in neutrophils, lymphocytes and eosinophils, except for monocytes in male rats. However, there was no significant difference in WBC count in either male or female rats, indicating that Zylaria does not impact the overall integrity of the immune system or cause tissue injury.

Additionally, PT and APTT are routinely measured to assess blood coagulation in general toxicological studies. In this study, there was an increase in PT and APTT in G4 and G4R compared to their respective vehicle control group. These changes observed in coagulation parameters could not be correlated with any changes in related parameters. These haematological findings further support the safety profile of Zylaria.

ALT, AST and ALP are sensitive markers for assessing liver function or liver injury [27]. Elevated activities of these enzymes are associated with liver damage [28]. In this study, the oral administration of Zylaria at a dosage up to 4500 mg/kg for 90 consecutive days demonstrated that AST, ALT and ALP levels of Zylaria‐treated rats were within the normal ranges, except lower levels of AST were noted in high doses of Zylaria‐treated female rats. However, the liver of female rats examined in the study was normal upon gross and histopathological examinations. This suggests that Zylaria is not hepatotoxic.

The serum total protein and albumin levels provide a useful indication of the nutritional status and functions of the liver and kidney [29], whereas serum urea, BUN and creatinine levels reflect the likelihood of renal problems or dysfunction [30]. Compared with the vehicle control group (G1), an insignificant difference in the serum level of these parameters was observed in Zylaria‐treated animals for 90 days; this further corroborates the fact that Zylaria does not cause liver damage and affects normal renal function.

Electrolytes such as Na, K and Cl play crucial roles in gas exchange and maintaining water balance between different body compartments. They are commonly used indicators to evaluate renal function [31]. Fluctuations in their serum levels can indicate either hypo‐ or hyperfunctioning of the kidneys [32]. In our study, we found no notable changes in Na, K and Cl levels in male rats treated with Zylaria. However, female rats exhibited a significant decrease in K levels despite histopathological examinations revealing normal kidney function. These findings suggest that Zylaria treatment does not adversely affect kidney function in rats.

Lipids play a vital role in maintaining proper physiological function within the body. However, an excess of lipids can elevate the risk of cardiovascular diseases. The lipid profile serves as a tool for monitoring cholesterol levels, including LDL‐cholesterol and HDL‐cholesterol, as well as triglycerides [33]. This study observed a decrease in total cholesterol, HDL cholesterol and LDL cholesterol in Zylaria‐treated animals. However, there was a significant difference in triglycerides in the mid‐dose of Zylaria‐treated female rats. The observed changes in lipid parameters were not dose‐dependent, were within physiological ranges and were not associated with any adverse pathological findings; therefore, they were considered non‐adverse and not toxicologically relevant.

The thyroid gland secretes thyroid hormones, which play a crucial role in controlling the overall metabolism of the body, protein synthesis, fat metabolism, neuronal and bone growth, and cardiovascular and renal functions [34]. In this study, low and mid doses of Zylaria‐treated male rats showed increased levels of T4. However, no change in absolute or relative thyroid weight and histopathological examinations were observed. This suggests a normal functioning status of the thyroid in Zylaria‐treated rats.

Increased organ weight (either absolute or relative) has been considered a sensitive indicator of organ toxicity by known toxicants [35]. Compared with the control group, an insignificant difference in the weight of the excised vital organs (e.g. liver, kidney, heart, brain, spleen, etc.) except the pituitary gland showed a statistical significance in mid‐ and high doses of Zylaria. However, no gross or histopathological changes were noted. This indicates that Zylaria on prolonged intake did not affect the normal functions of organs. As there was no reduction in relative organ weights in all Zylaria‐treated rats, it can be assumed that the extract is not toxic to these organs. From the results of the histological examination, no treatment‐related alterations or abnormalities were observed in the cell structure of organs. Although a few histopathological changes were observed in the sections of the liver, kidney, lungs, adrenal gland, testes, epididymides and ovaries, these changes were not treatment‐related because they were either incidental or spontaneous background changes commonly seen in this species and strain. Sporadic gross findings in the testes and epididymides are well documented as incidental background lesions in laboratory rats and, when observed at low incidence without dose dependency or consistent microscopic correlates, are not considered adverse or treatment‐related as supported by the literature stating ‘Tubular degeneration/atrophy, whether unilateral or bilateral, is a well‐recognised low‐incidence background finding in rats and mice’ [36]. Based on the observed results under the experimental conditions employed in the study, it is concluded that the no observed adverse effect level (NOAEL) of the test item Zylaria is equal to or greater than 4500 mg/kg bw/day.

## 5. Conclusions

Repeated administration of Zylaria to SD rats for 90 consecutive days had no test item–related effects on the general health of the animals, FOB parameters, clinical pathology parameters and pathology in both sexes. No test item–related effects were observed during the 28‐day recovery period after cessation of the treatment. Based on the observed results under the experimental conditions, it was concluded that Zylaria treatment for 90 days does not lead to toxicity, even at doses up to 4500 mg/kg bw/day, indicating its safety for use.

**Nomenclature**
A:G: Albumin–globulin ratio
AAALAC: American Association for Accreditation of Laboratory Animal Care
ALB: Albumin
ALP: Alkaline phosphatase
ALT: Alanine aminotransferase
ANOVA: Analysis of variance
APTT: Activated partial thromboplastin time
AST: Aspartate aminotransferase
BUN: Blood urea nitrogen
Ca: Calcium
CLW: C. chinensis water extract
CPCSEA: Committee for the Purpose of Control and Supervision of Experiments on Animals
CRE: Creatinine
FOB: Functional observation battery
GLUC: Glucose
HCT: Haematocrit
HGB: Haemoglobin
IAEC: Institutional Animal Ethics Committee
MCH: Mean corpuscular haemoglobin
MCHC: Mean corpuscular haemoglobin concentration
MCV: Mean corpuscular volume
MPV: Mean platelet volume
OECD: Organization for Economic Cooperation and Development
Phos: Phosphorus
PLT: Platelet
PT: Prothrombin time
RBC: Red blood cell
RDW: Red cell distribution width
SD: Sprague Dawley
TBA: Total bile acids
TBIL: Total bilirubin
TCHOL: Total cholesterol
TP: Total protein
WBC: White blood cell
XNE: X. nigripes
GLOB: Globulin
TRIG: Triglyceride
LDL: Low‐density lipoprotein cholesterol
HDL: High‐density lipoprotein cholesterol
URE: Serum urea
Na+: Sodium
K+: Potassium
Cl: Chloride
T3: Tri‐iodothyronine
T4: Thyroxine
TSH: Thyroid‐stimulating hormone

## Author Contributions

Richard Anthony Wang conceptualised the study, conducted data curation and contributed to the methodology. Devanand Shanmugasundaram managed project administration and contributed to data curation. Devanand Shanmugasundaram was responsible for visualisation and drafting the manuscript. Richard Anthony Wang and Devanand Shanmugasundaram reviewed and edited the manuscript. All the authors have accepted responsibility for the entire content of this manuscript.

## Funding

The study was funded by NuLiv Science USA Inc., Brea CA 92821.

## Disclosure

All authors approved the submission.

## Conflicts of Interest

RW is an employee of NuLiv Science and DS was an employee of Vedic Lifesciences Pvt. Ltd., and declare that there are no conflicts of interest. The authors and coauthors declare that there are no conflicts of interest regarding the publication of this article.

## Supporting Information

The Supporting file includes the extraction procedure for Zylaria, as well as additional tables covering feed consumption, coagulation parameters, urine parameters and gross necropsy.

Supporting Information for review and publication.

Figure 1: Histopathological image.

Supporting Figure S1a: Extraction procedure of Xylaria nigripes mycelium powder.

Supporting Figure S1b: Extraction procedure of Panax notoginseng extract. Supporting Figure S1c. Extraction procedure of Cuscuta chinensis extract. Supporting Figure S1d. Blending procedure of Xylaria nigripes mycelium powder Panax notoginseng extract and Cuscuta chinensis seed.

Supporting Figure S2a. High‐performance thin‐layer chromatography of Panax notoginseng root powder extract.

Supporting Figure S2b. High‐performance thin‐layer chromatography of Cuscuta Chinensis seed powder extract.

Supporting Figure S2c. High‐performance thin‐layer chromatography of ZylariaTM powder.

Supporting Figure S3a: Analytical report of total flavonoids %. Supporting Figure S3b: Analytical report of polysaccharides %. Supporting Figure S3c: Analytical report of γ aminobutyric acid mg/kg. Supporting Table S4: Feed consumption.

Supporting Table S5: Coagulation parameters. Supporting Table S6a: Urine parameters in males. Supporting Table S6b: Urine parameters in females. Supporting Table S7: Summary of gross necropsy findings.

## Supporting information


**Supporting Information** Additional supporting information can be found online in the Supporting Information section.

## Data Availability

The data that support the findings of this study are available from the corresponding author upon reasonable request.
