# Supplementary material for: 90‐Day Subchronic Toxicological Evaluation of a Zylaria in Sprague Dawley Rats
Source: J Toxicol. 2026 Apr 12;2026:6989296. doi: 10.1155/jt/6989296 (PMC13071335; doi:10.1155/jt/6989296)
Supplement: Supplementary file 1 — Supporting Information Additional supporting information can be found online in the Supporting Information section. [file JT-2026-6989296-s001.docx]

**Supplementary Figure S1a: Extraction procedure of** ***Xylaria nigripes* mycelium powder**

| Strain: *Xylaria nigripes* |
| --- |
| **↓** |
| Slant culture at 24~25°C for 4~5 days |
| **↓** |
| Shaking flask culture at 24~25°C for 8~9 days |
| **↓** |
| 5-liter tank culture at 24~25°C for 4~5 days |
| **↓** |
| 500-liter tank culture at 24~25°C for 4~5 days |
| **↓** |
| 60-ton tank culture at 24~25°C for 3~4 days |
| **↓** |
| Filter and separate |
| **↓** |
| Dry at 60°C for 2 days |
| **↓** |
| Dried extract |
| **↓** |
| Pulverize and pass through 100-mesh sieve |
| **↓** |
| Mix |
| **↓** |
| ***Xylaria nigripes* mycelium powder** |

**Supplementary Figure S1b: Extraction procedure of *Panax notoginseng* extract**

| *Panax notoginseng root* |
| --- |
| 1. Wash with water and dry at 80℃  2. Pulverize and screen through 20 mesh-size |
| Coarse granules |
| **↓** Extract with water at 80℃ |
| Liquid extract |
| **↓** Concentrate at 50℃ |
| Concentrated liquid extract |
| 1. Run through Diaion HP-20 absorptive resin column 2. Wash with water, discard eluted solution 3. Wash with 60% ethanol |
| Eluted solution |
| **↓** Concentrate at 50℃ |
| Concentrated liquid extract |
| **↓** Vacuum drying at 50℃ |
| Dried extract |
| **↓** Smash and screen thru 100 mesh-size |
| ***Panax notoginseng* extract** |

**Supplementary Figure S1c: Extraction procedure of *Cuscuta chinensis* extract**

| *Cuscuta chinensis* seed |
| --- |
| ↓ |
| Wash with 5-fold volume of purified water in ultrasonic machine for 40 min, three times |
| ↓ Discard liquid |
| Dry at 80C in vacuum for 5 hr |
| ↓ |
| Press degreasing |
| ↓ |
| Crush and pass through 14 mesh sieve |
| ↓ |
| Extract with 10-fold volume of 85% ethanol at 70C for 1hr, three times |
| ↓ |
| Filter |
| ↓ Discard solid |
| Liquid extract |
| ↓ |
| Concentrate at 80C in vacuum for 8 hr |
| ↓ |
| Spray drying |
| ↓ |
| Dried extract |
| ↓ |
| Pulverize and pass through 100-mesh sieve |
| ↓ |
| Mix |
| ↓ |
| ***Cuscuta chinensis* extract** |

**Supplementary Figure S1d: Blending procedure of *Xylaria nigripes mycelium* powder**

***Panax notoginseng* extract & *Cuscuta chinensis* seed**

| ***Xylaria nigripes mycelium* powder**  ***Panax notoginseng* extract**  ***Cuscuta chinensis* seed** |
| --- |
|  |
| 1. Blend and screen through 100 mesh-size 2. Pass through magnetic filter |
| Mixed powder |
| ↓ |
| Final Product (Zylaria powder) |

**Supplementary Figure S2a:** **High-Performance Thin-Layer Chromatography of *Panax notoginseng* Root Powder Extract**


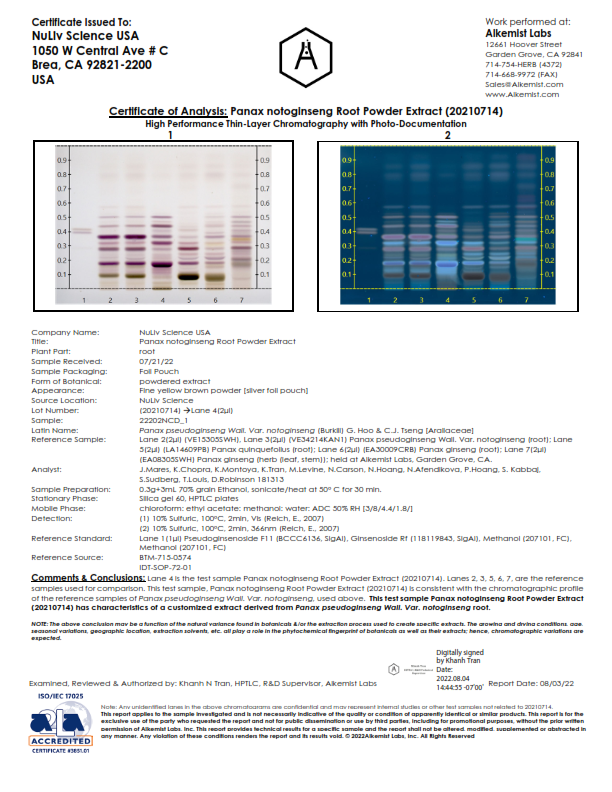


**Supplementary Figure S2b:** **High-Performance Thin-Layer Chromatography of *Cuscuta Chinensis* Seed Powder Extract**


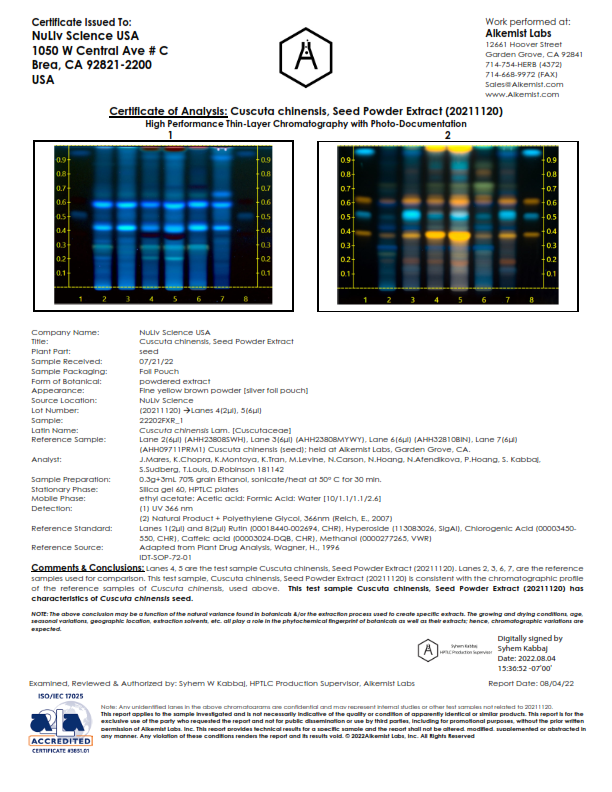


**Supplementary Figure S2c:** **High-Performance Thin-Layer Chromatography of Zylaria^TM^ Powder**


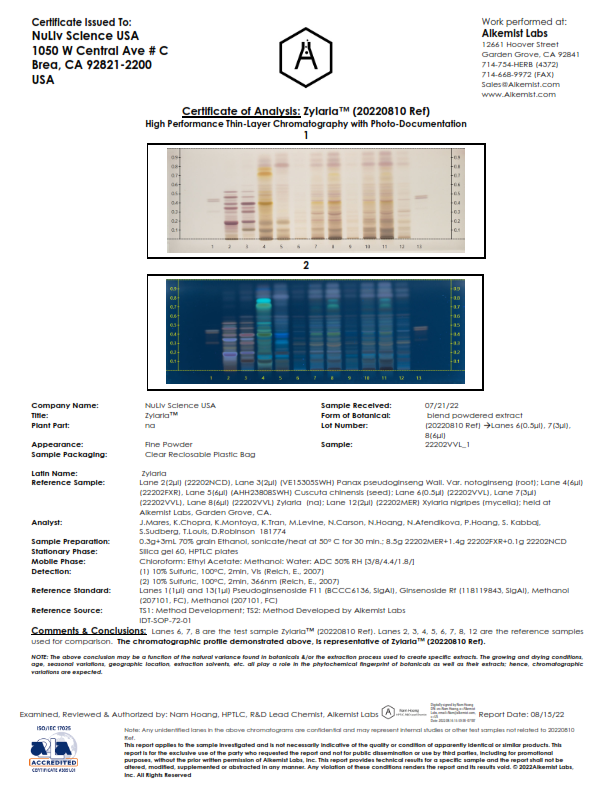


**Supplementary Figure S3a:** **Analytical Report of Total Flavonoids %**


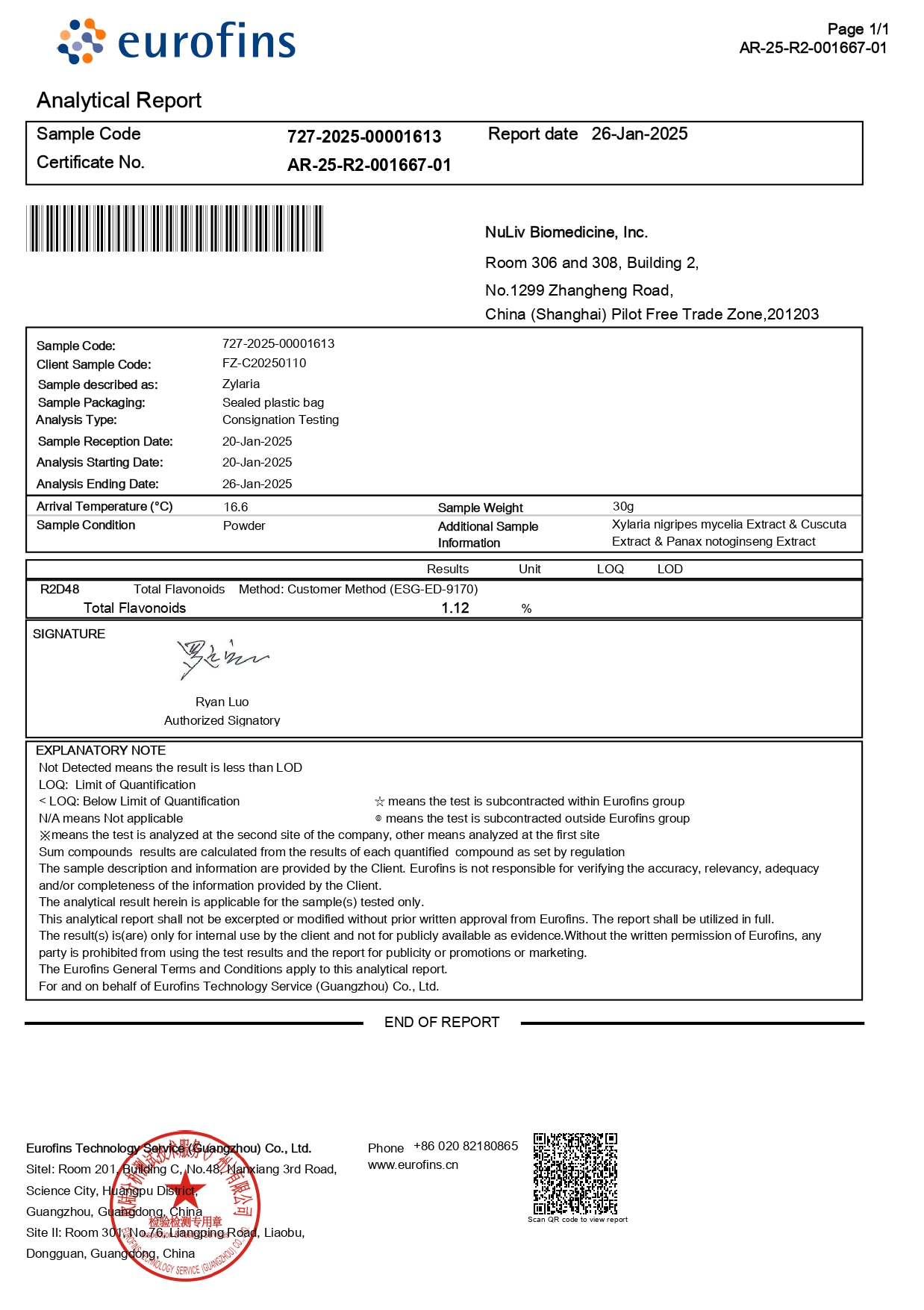


**Supplementary Figure S3b:** **Analytical Report of Polysaccharides %**


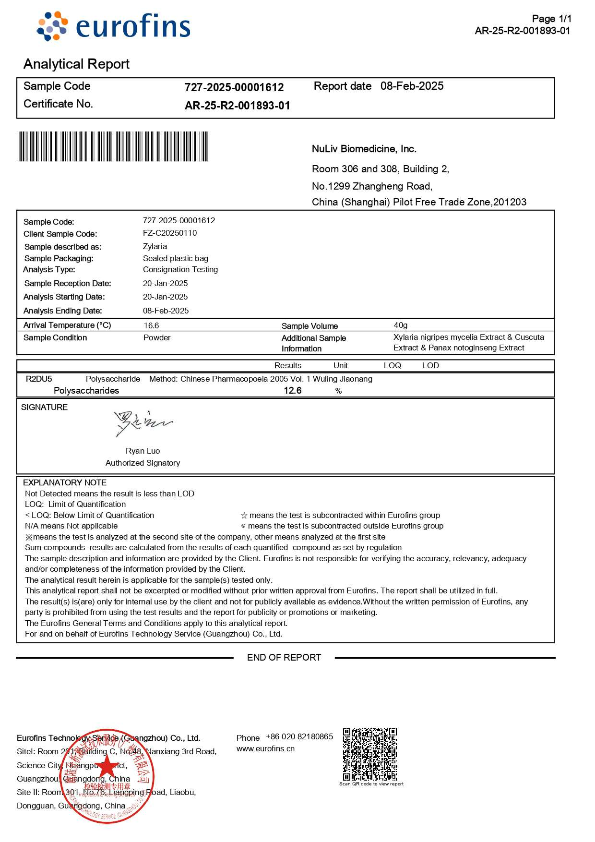


**Supplementary Figure S3c:** **Analytical Report of** γ **aminobutyric acid mg/kg**


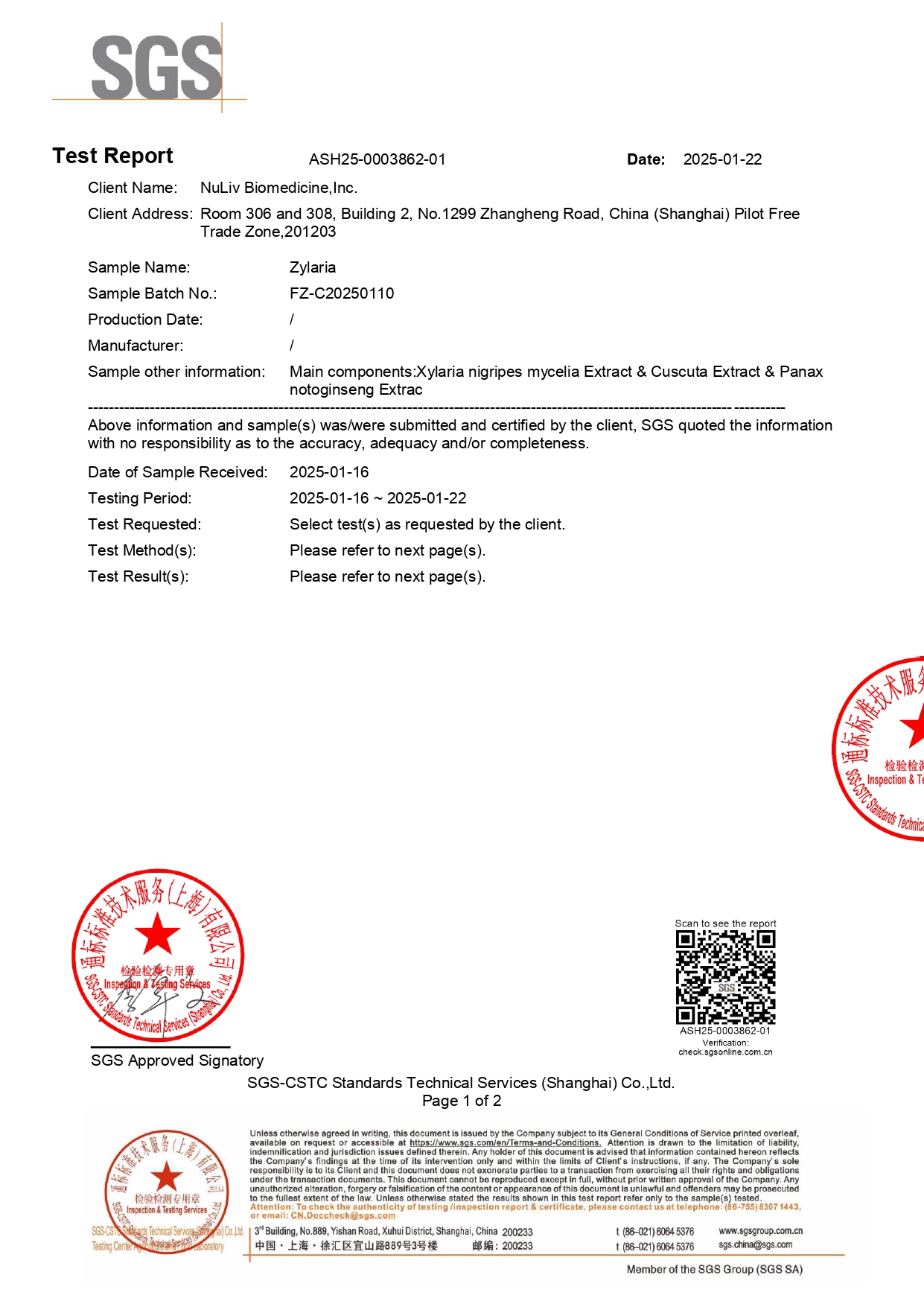


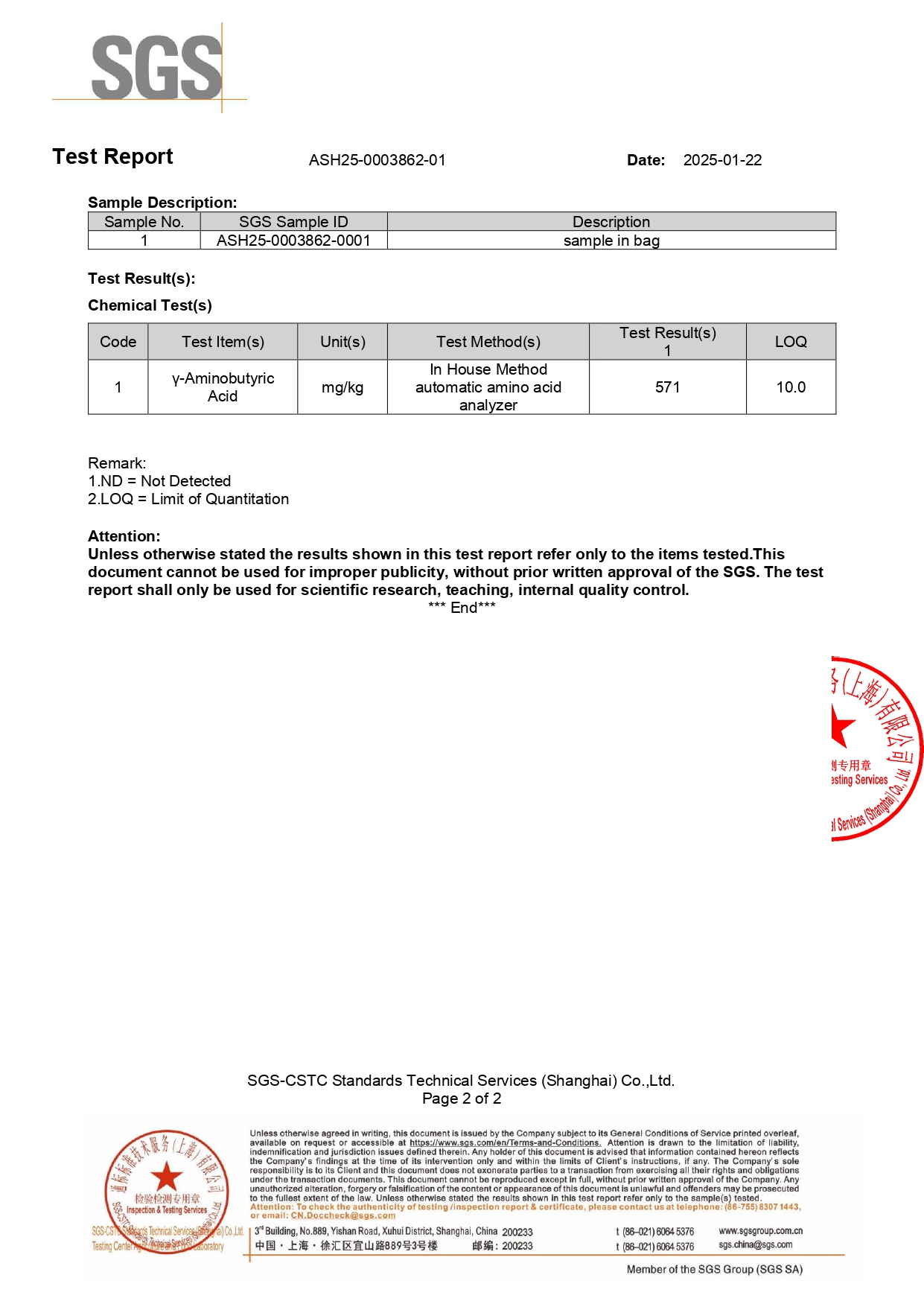


**Supplementary Table S4: Feed consumption**

| **Group** | | **G1** | **G2** | **G3** | **G4** | **G1R** | **G4R** |
| --- | --- | --- | --- | --- | --- | --- | --- |
| **Days** | **Sex** | **Vehicle control** | **Low dose Zylaria™** | **Mid dose Zylaria™** | **High dose Zylaria™** | **Vehicle control recovery** | **High dose of Zylaria™ recovery** |
| **8** | **Male** | 29.76±1.41 | 28.29±2.18 | 28.25±1.91 | 29.24±1.25 | 28.59±1.61 | 27.28±0.31 |
|  | **Female** | 19.23±1.39 | 19.03±1.11 | 18.48±0.84 | 19.27±2.58 | 17.19±1.96 | 17.61±0.10 |
| **15** | **Male** | 31.42±1.37 | 28.60±1.39 | 28.62±1.97 | 29.43±2.23 | 30.05±2.79 | 28.77±1.09 |
|  | **Female** | 20.28±2.11 | 20.12±0.64 | 19.98±1.12 | 20.88±2.27 | 18.20±2.45 | 19.94±0.65 |
| **22** | **Male** | 33.17±2.24 | 30.79±1.31 | 30.73±1.45 | 31.02±1.42 | 31.24±4.18 | 28.27±0.10 |
|  | **Female** | 21.91±2.56 | 21.13±1.77 | 20.95±1.55 | 20.92±1.63 | 19.59±1.73 | 20.87±0.19 |
| **29** | **Male** | 33.46±1.75 | 31.26±1.60 | 30.22^a^±2.06 | 31.65±2.38 | 34.23±2.05 | 29.38±2.73 |
|  | **Female** | 21.99±2.87 | 21.39±0.96 | 20.64±1.08 | 20.44±2.04 | 19.96±1.39 | 21.13±1.18 |
| **36** | **Male** | 33.26±1.75 | 31.38±1.55 | 29.99^a^±2.40 | 32.12±1.79 | 32.41±0.23 | 28.79±1.45 |
|  | **Female** | 21.87±3.24 | 20.99±1.19 | 20.06±1.78 | 21.02±1.76 | 20.62±0.91 | 21.46±0.89 |
| **43** | **Male** | 33.99±2.50 | 31.79±2.37 | 31.42±2.28 | 33.15±1.65 | 33.85±1.43 | 31.54±0.81 |
|  | **Female** | 20.83±2.28 | 21.11±2.31 | 20.67±1.78 | 21.40±0.83 | 20.75±1.21 | 21.45±0.17 |
| **50** | **Male** | 34.78±2.26 | 33.04±2.00 | 32.55±2.80 | 33.65±2.64 | 33.55±1.35 | 31.28±0.26 |
|  | **Female** | 22.43±1.81 | 22.41±1.63 | 21.11±1.41 | 21.36±1.06 | 21.50±1.85 | 23.20±1.63 |
| **57** | **Male** | 34.44±2.52 | 32.47±2.12 | 30.49±2.23 | 32.49±2.83 | 33.57±1.31 | 31.32±1.33 |
|  | **Female** | 22.50±2.93 | 21.52±2.25 | 21.23±1.39 | 22.57±2.93 | 21.03±1.51 | 21.81±2.32 |
| **64** | **Male** | 30.97±2.09 | 30.90±2.07 | 29.76±2.47 | 30.27±2.41 | 32.15±0.00 | 30.29±0.91 |
|  | **Female** | 22.00±2.24 | 21.68±2.03 | 21.16±1.55 | 21.38±1.47 | 20.87±0.43 | 22.02±0.85 |
| **71** | **Male** | 31.43±2.44 | 31.05±2.84 | 30.51±2.44 | 31.67±2.81 | 31.89±1.07 | 30.30±0.78 |
|  | **Female** | 22.05±1.59 | 22.03±2.06 | 21.63±1.41 | 22.08±1.03 | 19.34±1.38 | 21.50±0.58 |
| **78** | **Male** | 31.25±2.51 | 31.27±2.50 | 30.24±2.56 | 32.15±2.69 | 31.71±0.51 | 29.33±0.32 |
|  | **Female** | 21.95±2.10 | 21.39±2.63 | 22.04±1.79 | 21.88±1.89 | 20.58±3.15 | 23.09±4.30 |
| **85** | **Male** | 30.18±2.53 | 30.61±1.96 | 29.69±1.95 | 32.10±1.65 | 32.44±0.77 | 30.09±0.77 |
|  | **Female** | 21.99±2.50 | 21.55±1.84 | 21.36±1.51 | 22.25±3.68 | 19.80±2.76 | 25.08±5.91 |
| **90** | **Male** | 31.26±3.53 | 32.17±2.91 | 32.13±4.36 | 33.87±3.41 | 28.92±1.50 | 28.44±0.19 |
|  | **Female** | 20.87±2.21 | 21.08±1.54 | 21.25±2.61 | 17.66±1.26 | 19.39±2.14 | 19.73±1.84 |
| **99** | **Male** | - | - | - | - | 33.13±0.55 | 32.70±1.54 |
|  | **Female** | - | - | - | - | 23.11±1.04 | 25.60±4.89 |
| **106** | **Male** | - | - | - | - | 27.01±2.20 | 29.84±4.57 |
|  | **Female** | - | - | - | - | 17.16±0.08 | 19.59±3.54 |
| **113** | **Male** | - | - | - | - | 31.21±0.09 | 29.20±0.71 |
|  | **Female** | - | - | - | - | 19.53±3.22 | 19.35±2.09 |
| **118** | **Male** | - | - | - | - | 32.40±0.89 | 30.66±0.09 |
|  | **Female** | - | - | - | - | 19.91±2.70 | 20.10±0.12 |

Note: Values as mean ± SD (main groups treated for 90 days, n=10 each; recovery groups, n=5); ^a^Indicates statistically significant change at p<0.05 compared to vehicle control

**Supplementary Table S5: Coagulation parameters**

| **Group** | | **Coagulation Parameters (sec)** | | | |
| --- | --- | --- | --- | --- | --- |
|  |  | **Male** | | **Female** | |
|  |  | **PT** | **APTT** | **PT** | **APTT** |
| **G1** | **Vehicle Control** | 10.94±1.35 | 16.26±2.66 | 11.37±1.24 | 18.19±3.64 |
| **G2** | **Low dose Zylaria™** | 12.63±2.49 | 16.67±2.90 | 12.02±2.04 | 16.79±1.54 |
| **G3** | **Mid dose Zylaria™** | 12.50±1.91 | 16.53±2.78 | 11.36±0.79 | 15.39±1.40 |
| **G4** | **High dose Zylaria™** | 14.74^a^±1.60 | 16.67±3.18 | 12.46±1.91 | 17.97±2.82 |
| **G1R** | **Vehicle control recovery** | 11.58±2.15 | 17.28±1.40 | 10.84±0.96 | 16.36±0.87 |
| **G4R** | **High dose of Zylaria™ recovery** | 11.78±2.02 | 16.74±2.34 | 11.80±3.29 | 18.70^a^±2.05 |

Note: Values are Mean ± SD for main groups (n=10) and recovery (n=5) groups. ^a^Indicate statistically significant change at p<0.05 in comparison to respective vehicle control and recovery control vehicle group.

**Supplementary Table S6a: Urine parameters in males**

| **Groups** | | **G1** | **G2** | **G3** | **G4** | **G1R** | **G4R** |
| --- | --- | --- | --- | --- | --- | --- | --- |
| **Parameters** | | **Vehicle control** | **Low dose Zylaria™** | **Mid dose Zylaria™** | **High dose Zylaria™** | **Vehicle control recovery** | **High dose of Zylaria™ recovery** |
| **Volume (mL)** | Mean | 16.00±7.38 | 15.00±5.77 | 15.50±5.99 | 11.00±5.16 | 11.00±2.24 | 9.00±2.24 |
| **Appearance** | Clear | 9 | 8 | 10 | 8 | 5 | 4 |
|  | Slightly turbid | 1 | 2 | 0 | 2 | 0 | 1 |
| **Specific Gravity** | Mean | 1.02±0.00 | 1.02^a^±0.00 | 1.02±0.00 | 1.02±0.00 | 1.02±0.00 | 1.02±0.00 |
| **pH** | Mean | 5.55±0.72 | 5.80±1.48 | 5.55±0.76 | 5.85±0.82 | 5.40±0.55 | 5.60±0.55 |
| **WBC** | Neg | 8 | 8 | 9 | 8 | 4 | 1 |
|  | 25 | 2 | 0 | 1 | 2 | 1 | 3 |
|  | 100 | 0 | 1 | 0 | 0 | 0 | 1 |
|  | 500 | 0 | 1 | 0 | 0 | 0 | 0 |
| **Protein (mg/dL)** | Neg | 10 | 8 | 10 | 8 | 5 | 5 |
|  | 25 | 0 | 2 | 0 | 2 | 0 | 0 |
| **Glucose (mg/dL)** | Norm | 10 | 10 | 10 | 10 | 5 | 5 |
| **Erythrocytes** | Neg | 10 | 9 | 9 | 7 | 5 | 5 |
|  | 10 | 0 | 1 | 1 | 3 | 0 | 0 |
|  | 25 | - | - | - | - | 0 | 0 |
| **Nitrite** | Neg | 7 | 7 | 6 | 3 | 5 | 5 |
|  | Pos | 3 | 3 | 4 | 7 | 0 | 0 |
| **Ketone bodies (mg/dL)** | Neg | 10 | 9 | 10 | 9 | 5 | 5 |
|  | 5 | 0 | 0 | 0 | 1 | 0 | 0 |
|  | 15 | 0 | 1 | 0 | 0 | - | - |
| **Urobilinogen** | Normal | 10 | 10 | 10 | 10 | 5 | 5 |
| **Bilirubin (mg/dL)** | Neg | 10 | 10 | 10 | 10 | 5 | 5 |
| **Color** | Pale Yellow | 10 | 9 | 10 | 9 | 5 | 5 |
|  | Yellow | 0 | 1 | 0 | 0 | - | - |
|  | Amber | 0 | 0 | 0 | 1 | - | - |

Note: Values are Mean ± SD for main (n=10) treated for 90 days and recovery (n=5) groups. ^a^Indicate statistically significant change at p<0.05 when compared with the vehicle control group.

**Supplementary Table S6b: Urine parameters in females**

| **Groups** | | **G1** | **G2** | **G3** | **G4** | **G1R** | **G4R** |
| --- | --- | --- | --- | --- | --- | --- | --- |
| **Parameters** | | **Vehicle control** | **Low dose Zylaria™** | **Mid dose Zylaria™** | **High dose Zylaria™** | **Vehicle control recovery** | **High dose of Zylaria™ recovery** |
| **Volume (mL)** | Mean | 14.00±3.94 | 13.50±5.80 | 12.00±4.22 | 8.50^a^±3.37 | 8.00±2.74 | 7.00±2.74 |
| **Appearance** | Clear | 9 | 7 | 7 | 6 | 3 | 2 |
|  | Slightly turbid | 1 | 3 | 3 | 4 | 2 | 3 |
| **Specific Gravity** | Mean | 1.02±0.01 | 1.02±0.01 | 1.02±0.01 | 1.02±0.00 | 1.02±0.00 | 1.02±0.00 |
| **pH** | Mean | 6.50±1.35 | 6.95±1.07 | 7.05±1.01 | 6.90±0.99 | 6.40±1.67 | 6.80±1.10 |
| **WBC** | Neg | 8 | 3 | 3 | 0 | 3 | 2 |
|  | 25 | 0 | 4 | 2 | 3 | 0 | 0 |
|  | 100 | 2 | 3 | 3 | 3 | 1 | 1 |
|  | 500 | 0 | 0 | 2 | 4 | 1 | 2 |
| **Protein (mg/dL)** | Neg | 9 | 7 | 7 | 5 | 3 | 3 |
|  | 25 | 1 | 3 | 3 | 5 | 2 | 2 |
| **Glucose (mg/dL)** | Norm | 10 | 10 | 10 | 10 | 5 | 5 |
| **Erythrocytes** | Neg | 8 | 8 | 9 | 10 | 4 | 5 |
|  | 10 | 2 | 1 | 1 | 0 | 1 | 0 |
|  | 25 | 0 | 0 | 0 | 0 | - | - |
|  | 50 | 0 | 1 | 0 | 0 | 0 | 0 |
| **Nitrite** | Neg | 8 | 8 | 8 | 9 | 5 | 5 |
|  | Pos | 2 | 2 | 2 | 1 | 0 | 0 |
| **Ketone bodies (mg/dL)** | Neg | 9 | 7 | 10 | 8 | 4 | 3 |
|  | 5 | 1 | 3 | 0 | 2 | 1 | 1 |
|  | 15 | 0 | 0 | 0 | 0 | 0 | 1 |
| **Urobilinogen** | Normal | 10 | 10 | 10 | 10 | 5 | 5 |
| **Bilirubin (mg/dL)** | Neg | 9 | 9 | 7 | 5 | 5 | 5 |
|  | 1 | 1 | 1 | 3 | 5 | - | - |
| **Color** | Pale Yellow | 9 | 6 | 7 | 5 | 5 | 5 |
|  | Yellow | 0 | 1 | 2 | 1 | - | - |
|  | Amber | 1 | 3 | 1 | 4 | - | - |

Note: Values are Mean ± SD for main groups (n=10) and recovery groups (n=5). ^a^Indicate statistically significant change at p<0.05 when compared with the vehicle control group.

**Supplementary Table S7: Summary of Gross Necropsy Findings**

| **Group** | | **G1** | **G2** | **G3** | **G4** | **G1R** | **G4R** |
| --- | --- | --- | --- | --- | --- | --- | --- |
| **Observation** | **Sex** | **Vehicle control** | **Low dose Zylaria™** | **Mid dose Zylaria™** | **High dose Zylaria™** | **Vehicle control recovery** | **High dose of Zylaria™ recovery** |
| No Abnormality Detected | Male | 10/10 | 10/10 | 10/10 | 8/10 | 5/5 | 5/5 |
|  | Female | 10/10 | 10/10 | 10/10 | 10/10 | 5/5 | 5/5 |
| Testes- Small size; Unilateral/bilateral | Male | - | - | - | 1/10 | - | - |
| Epididymides (bilateral) – Small sized |  | - | - | - | 1/10 | - | - |
